# Breast Cancer Stem-Like Cells in Drug Resistance: A Review of Mechanisms and Novel Therapeutic Strategies to Overcome Drug Resistance

**DOI:** 10.3389/fonc.2022.856974

**Published:** 2022-03-21

**Authors:** Taniya Saha, Kiven Erique Lukong

**Affiliations:** Department of Biochemistry, Microbiology and Immunology, University of Saskatchewan, Saskatoon, SK, Canada

**Keywords:** breast cancer, drug resistance, BCSCs, miRNAs, therapeutic strategy, cancer stem-like cells

## Abstract

Breast cancer is the most frequent type of malignancy in women worldwide, and drug resistance to the available systemic therapies remains a major challenge. At the molecular level, breast cancer is heterogeneous, where the cancer-initiating stem-like cells (bCSCs) comprise a small yet distinct population of cells within the tumor microenvironment (TME) that can differentiate into cells of multiple lineages, displaying varying degrees of cellular differentiation, enhanced metastatic potential, invasiveness, and resistance to radio- and chemotherapy. Based on the expression of estrogen and progesterone hormone receptors, expression of human epidermal growth factor receptor 2 (HER2), and/or BRCA mutations, the breast cancer molecular subtypes are identified as TNBC, HER2 enriched, luminal A, and luminal B. Management of breast cancer primarily involves resection of the tumor, followed by radiotherapy, and systemic therapies including endocrine therapies for hormone-responsive breast cancers; HER2-targeted therapy for HER2-enriched breast cancers; chemotherapy and poly (ADP-ribose) polymerase inhibitors for TNBC, and the recent development of immunotherapy. However, the complex crosstalk between the malignant cells and stromal cells in the breast TME, rewiring of the many different signaling networks, and bCSC-mediated processes, all contribute to overall drug resistance in breast cancer. However, strategically targeting bCSCs to reverse chemoresistance and increase drug sensitivity is an underexplored stream in breast cancer research. The recent identification of dysregulated miRNAs/ncRNAs/mRNAs signatures in bCSCs and their crosstalk with many cellular signaling pathways has uncovered promising molecular leads to be used as potential therapeutic targets in drug-resistant situations. Moreover, therapies that can induce alternate forms of regulated cell death including ferroptosis, pyroptosis, and immunotherapy; drugs targeting bCSC metabolism; and nanoparticle therapy are the upcoming approaches to target the bCSCs overcome drug resistance. Thus, individualizing treatment strategies will eliminate the minimal residual disease, resulting in better pathological and complete response in drug-resistant scenarios. This review summarizes basic understanding of breast cancer subtypes, concept of bCSCs, molecular basis of drug resistance, dysregulated miRNAs/ncRNAs patterns in bCSCs, and future perspective of developing anticancer therapeutics to address breast cancer drug resistance.

## Introduction

Breast cancer (BC) is the most frequent type of malignancy in women worldwide. BC has now eclipsed lung cancer as the leading cause of global cancer incidence in 2020, with an estimated 2.3 million new cases, representing 11.7% of all cancer cases (1). Drug resistance in BC patients appears to be the major challenge in breast cancer research. Despite significant advances in BC treatment, many patients with malignant BC experience aggressive disease progression due to *de novo* and acquired drug resistance. *De novo* resistance occurs even before drug exposure, while acquired resistance emerges from initially drug-sensitive tumors. The mechanisms associated with *de novo* drug resistance significantly contribute to failure to eradicate the residual disease, thus facilitating the development of acquired drug resistance (2).

The failure of the current treatment therapies as well as the high mortality in metastatic BC patients is highly attributed to the existence of therapy-resistant breast cancer stem-like cells (bCSCs). The emerging concept of CSC origin supports the hierarchical organization of CSC-like cells, sitting at the top of the hierarchy, having the unique ability to give rise to diverse lineages of cancer cells that forms the tumor. Although the CSC-like cells occupy only a very minor fraction of the total tumor mass (around 2%), they are mainly responsible for establishing the intratumor heterogeneity (3). The concept of “tumor heterogeneity” refers to the genetic variation existing between tumor cells within and across the BC patients. According to the CSC concept, the CSC-like cells possess three main characteristics: (1) potent tumor initiation potential to regenerate the tumor, (2) self-renewal feature *in vivo* that would inevitably allow them to form a phenotypically indistinguishable heterogeneous tumor, when transplanted in secondary or tertiary recipients, and (3) finally, they must reflect the differentiation ability so that they can re-establish a phenocopy of the original tumor. Hajj et al. first identified and isolated the bCSCs (CD44+CD24−/lowLineage−) from the phenotypically diverse population of BC cells. This fraction of breast tumor cells can form a new tumor with additional CD44+CD24−/lowLineage− bCSCs along with phenotypically different nontumorigenic cells (4). If the therapy in question fails to specifically target and kill the bCSCs, they would persist as the residual disease, which can regenerate tumors in the future. Frequently, the bCSCs overexpress the drug efflux transporters and spend most of the time in the nondividing cell-cycle phase (G0) to escape from the conventional therapeutics (5). Hence, targeting the bCSCs in any subtype, such as luminal A, luminal B, human epidermal growth factor receptor 2 (HER2)-enriched, and triple-negative (TNBC), is the key strategy to conquer therapeutic resistance in BC. The complex communication between bCSCs and the stromal cells; resistance to chemotherapeutic drugs (paclitaxel, anthracycline, platinum), endocrine therapies (tamoxifen, fulvestrant), and HER2-targeted drugs (trastuzumab, lapatinib); rewiring of hedgehog (Hh), Notch, Wnt/β-catenin, and phosphoinositide 3-kinase (PI3K)/Akt/mTOR signaling networks; and enhancing DNA repair mechanism, contribute to the overall drug resistance in bCSCs (6).

MicroRNAs (miRNAs) also add another dimension to the complexity of BC disease progression and therapeutic resistance, through maintenance of the bCSC population. MiRNAs are a group of small noncoding RNAs (ncRNAs) that influence the expression of their target genes at the posttranscriptional level by binding to the 3′-untranslated regions (3′-UTR) of mature mRNA transcripts. Two different types of miRNAs—tumor suppressor miRNAs (miR34, Let-7, miR30, miR200 family, miR600) and Onco-miRs (miR-22, miR155, miR181, miR221/222 cluster)—have been identified in bCSCs, having either tumor-suppressive or oncogenic functions, respectively. Interestingly, miRNAs have been implicated in the regulation of many different signaling networks, contributing to the development and maintenance of bCSCs (3, 7). Moreover, locoregional tumor burden along with the metastatic patterns in BC patients also influence the efficacy of the treatment strategies. In the case of early-stage BC, the tumor is restricted in the breast or local axillary lymph node, and the success rate for relapse-free survival is around 70%–80% (8). However, in the case of advanced BC, where metastatic dissemination from the primary tumor site leads to the re-establishment of secondary tumors involving other organs like lung, brain, liver, and bones, complete cure is not possible. In that scenario, much emphasis is given to prolonging the patient survival to exert a low degree of treatment-associated cytotoxicity to improve the quality of life.

Due to the lack of specific molecular targets in TNBC and increased resistance to the anti-HER2 therapies in HER2+ breast tumors, cytotoxic chemotherapy is the common alternative for treating these two most resistant subtypes of BC. However, there is an increasing search for therapeutic strategies that would sensitize the drug-resistant bCSCs to programmed cell death. Immunotherapy, based on the immune checkpoint inhibitor molecules (ICIs), specific for programmed cell death protein 1 (PD-1), programmed death ligand 1 (PD-L1), and CTLA-4, which are either administered as a single agent or in combination with either a humanized monoclonal antibody, such as trastuzumab, in HER2+ BC settings, or any other chemotherapeutic drug in the TNBC scenario, have been the recent candidates in clinical trials (8). Other immunotherapy approaches like the chimeric antigen receptor T-cell (CAR-T) therapy, dendritic cell (DC) vaccine, and oncolytic viral therapies, specific for bCSC immune targeting, are also gaining significant momentum in recent years (9). Nanoparticle-based bCSC-targeting platform is also appearing as an upcoming approach to deliver small molecules, antibodies, and miRNAs to affect the signaling networks implicated in bCSC self-renewal and differentiation; interfering with drug-efflux transporters; and targeting bCSC metabolism (10). In this review, we focused on the mechanisms of resistance to chemo-, endocrine, and targeted therapies, the contribution of bCSCs in exerting drug resistance, and the factors influencing bCSC-mediated drug resistance and, finally, we emphasized the alternative forms of upcoming treatment platforms to overcome CSC-related drug resistance in BC patients.

## Breast Architecture, BC Subtypes, and Advancement of Technologies to Identify BC

**Figure 1:**
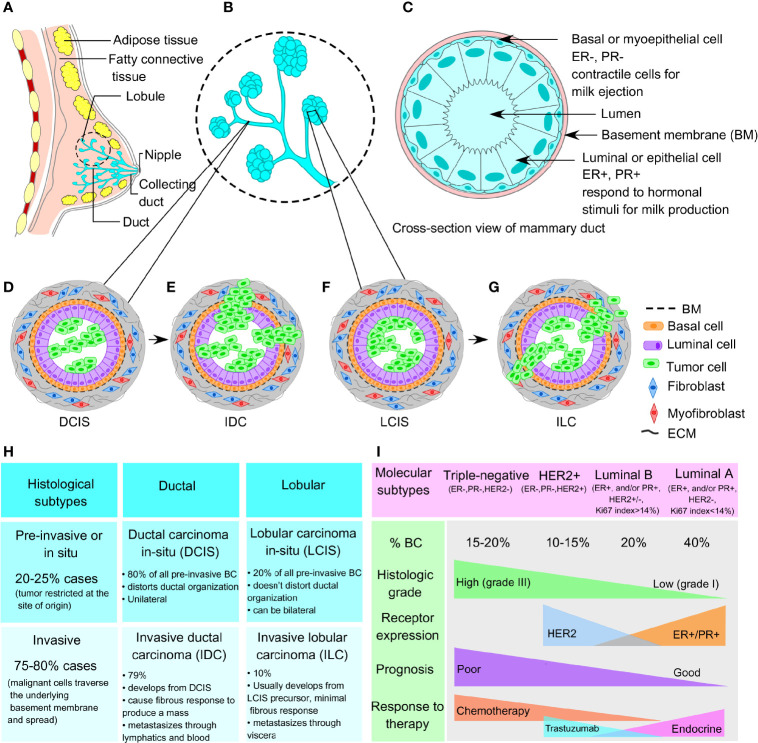
Normal breast architecture and breast cancer subtypes. **(A)** Representative image of breast architecture showing lobular and ductal system. **(B)** Magnified view of milk duct showing detailed lobular and ductal structure as an inset image. **(C)** Cross-sectional view of normal mammary duct showing basement membrane, basal myoepithelial cell layer, and luminal or epithelial cell layer from outside to inside. **(D)** Representative images of ductal carcinoma *in situ* (DCIS) and **(E)** invasive ductal carcinoma (IDC). **(F)** Representative images of lobular carcinoma *in situ* (LCIS) and **(G)** invasive lobular carcinoma (ILC). **(H)** Histological subtypes (preinvasive and invasive) and **(I)** molecular subtypes (triple-negative, HER2+, luminal A, and luminal B) of breast cancer.

The breast architecture mainly involves glandular tissue including the breast lobes and ducts, supportive fibrous connective tissue, and fatty tissues that largely fill in the gaps between the glandular and fibrous tissues. An adult woman’s breast consists of 15–20 lobes, each lobe further containing 20–40 lobules. The lobules resemble a grape-like structure, where each of the lobules is attached to a small milk duct, and finally these small ducts join, eventually forming a larger collecting duct. There are around 10 ductal systems present in each breast that finally open at the nipple. A cross-section of a milk duct shows a basement membrane layer, the basal or myoepithelial layer, and the luminal or epithelial layer, from outside to inside (**Figures 1A–C**). In 20%–25% of cases, the tumor is restricted at the site of its origin (*in situ* or preinvasive); whereas, in 75%–80% of cases, the tumors are malignant (invasive) whereby the malignant cells invade the basement membrane and penetrate the stroma.

### Histological and Molecular Subtypes

BCs are generally divided according to histological grade and stage, which define the aggressiveness and metastatic potential of the tumor. Histologically, the breast tumors are distinguished into preinvasive and invasive subtypes involving the ductal and lobular compartments. Ductal and lobular subtype is classified as ductal carcinoma *in situ* (DCIS), invasive ductal carcinoma (IDC), lobular carcinoma *in situ* (LCIS), and invasive lobular carcinoma (ILC), respectively (**Figures 1D–H**) (11). At the molecular level, breast tumors are categorized into 4 main subtypes, based on the presence/absence of markers that include estrogen receptors (ER), progesterone receptors (PR), and HER2, as well as their proliferative index according to Ki67 expression (12). These molecular subtypes include TNBC (ER−, PR−, HER2−), HER2-enriched (ER−, PR−, HER2+), luminal A (ER+ and/or PR+, but HER2−, Ki67 <14%), and luminal B (ER+ and/or PR+, HER2+ or HER2−, Ki67 >14%) (refer to **Figure 1I**). ER+ breast tumors are targeted using selective estrogen receptor modulators (SERMs), aromatase inhibitors, cyclin-dependent kinases 4 and 6 (CDK4/6) inhibitors, and ER degraders also called selective estrogen receptor downregulators (SERDs) (13). HER2-enriched breast tumors are candidates for HER2-targeted monoclonal antibodies. However, TNBC accounts for the most therapy-resistant subtype of heterogeneous basal-like tumors (15%–20% of all breast tumors), which frequently reflects a high mutational burden including tumor suppressor p53 (TP53 gene, 74.5%–82.8%), breast cancer type-1 and/or type-2 susceptibility gene (BRCA1, 1.96%–21.55%; BRCA2, 1.63%–18.10%), and phosphatidylinositol 3-kinase catalytic alpha polypeptide (PI3KCα, 8.6%–23.2%) (9, 14–16). However, another compelling piece of evidence from Maristany et al. suggests the concept of phenotypic switching between BC molecular subtypes, as evident from the gene expression studies before, during, and after neoadjuvant therapy with lapatinib and trastuzumab in HER2+/HER2-enriched tumors of the PAMELA trial and BC cell lines (17). Dual blockade of HER2 pathway in HER2-enriched settings leads to a subtype switching to a low-proliferative luminal A phenotype both in the patients’ tumor samples and *in vitro* models. Strikingly, this subtype switching from HER2-enriched to luminal A phenotype increased the sensitivity toward CDK4/6 inhibitors; although, this switching is reversible upon stopping the anti-HER2 treatment. Moreover, integrated analysis of copy number and gene expression studies of 2,000 breast tumors by Curtis et al. reveals the existence of novel molecular stratification among the BC population, resulting from the impact of somatic copy number aberrations (CNAs) on the transcriptome (18). A similar study of somatic CNAs revealed an advanced stratification of BC cases into integrative clusters and prototypical patterns of single-nucleotide variants, shaping the clinical courses and response to BC therapies (19). Therefore, in addition to the conventional BC molecular subtypes discussed above, the genomic and transcriptomic architecture of BC samples further add another dimension, yielding novel BC subgroups with distinct clinical outcomes. Moreover, the emerging evidence on the inter- and intratumoral heterogeneity within a breast tumor not only acknowledges the probability that multiple different BC subtypes can coexist within a single tumor but also demonstrates that plasticity between divergent subtypes is possible rather than being static (20). Interconversion between the different subtypes within a breast tumor contributes to disease progression, metastasis, and therapeutic resistance. Therefore, therapeutic decision making must be designed based on the genomic and transcriptomic inputs along with the changing molecular phenotypes even in an individual patient’s tumor.

### Solid Tissue Biopsy and Liquid Biopsy

Solid tissue biopsy is the standard method of choice in clinical oncology that provides information on tumor histology, molecular profiling and subtyping, and biomarkers targeted for treatment planning (21). However, it does not reveal the complete genomic landscape of the tumor, as the tissue is collected from a specific biopsy area. The heterogeneous nature of the tumor is validated when tumor cells collected from multiple regions from a single patient are subjected to exome sequencing, ploidy profiling, and chromosome aberration analysis. Gerlinger et al., for example, noted that approximately 63%–69% of the mutations observed in tumor tissue obtained from a single biopsy derived from the same patient are not homogeneous throughout the tumor. This observation strongly indicates the importance of “multiregion biopsy” for the diagnosis of cancer (22).

To bypass this limitation, in 2020, the Food and Drug Administration (FDA) approved the use of “liquid biopsy” where DNA from circulating breast tumor cells (ctDNA) shed from the primary tumor site is isolated from the patient’s blood and then subjected to microfluidic-based single-cell transcriptional profiling (23, 24). CtDNAs released into the systemic circulation can be theoretically defined as an admixture of tumor DNA samples from different metastatic sites, thus fully reflecting the tumor heterogeneity. A very recent work from Kingston et al. represents the application of plasma ctDNA sequencing to define the genomic profile of metastatic BC in 800 patients in the plasmaMATCH trial (25). With this novel approach, diverse resistance mutations including enrichment of HER2 mutations in HER2+ tumors, ESR1 and MAPK pathway mutations in ER+ HER2− tumors, and multiple PI3KCA mutations in ER+ tumors, have been successfully demonstrated. Particularly, this study utilizes the ctDNA analysis platform in a large clinical trial to denote the subclonal diversification of pretreated advanced BC, categorizing unique mutational processes in ER+ BC and identifying novel therapeutic directions. This noninvasive methodology enables the detection of early stages of BC, monitoring of treatment efficacy and therapeutic resistance, and identification of minimum residual disease (MRD) and risk of relapse (24, 26). Circulating tumor RNAs are also released into the bloodstream of BC patients, which provides another analytic platform through the liquid biopsy method (27). Next-generation sequencing (NGS) is also increasingly applied for high-throughput BC mutational profiling (28, 29).

## bCSCs and Drug Resistance

BCSCs can escape the conventional therapies through adaptation to several strategies, where the breast cancer stem-like cells can remain dormant or “quiescent”, turn off the apoptotic pathways, and increase DNA repair mechanisms, along with expelling chemotherapy (chemo) drugs out of the cell, manipulating TME, and managing the intracellular load of reactive oxygen and nitrogen species (ROS and RNS) (30). Although the dormant bCSCs maintain themselves in the G0 state, they still retain the ability to enter the cell division cycle in response to mitotic stimulation (31). As chemotherapy and radiation therapy exclusively target the proliferating fraction of tumor cells, the bCSCs can evade systemic therapies, and in turn, develop drug resistance. Thus, drug resistance confers the bCSCs with a selective advantage over the non-CSCs that supports the “survival of the fittest” hypothesis applicable for CSC-like cells within TME. Moreover, epithelial–mesenchymal transition (EMT) plasticity that enables bCSCs to dynamically switch between intermediate cellular states of varying epithelial/mesenchymal traits, also contributes to bCSC-mediated therapeutic resistance (32–34). A study by Liu et al. demonstrates that bCSCs exhibit plasticity that allows them to transition between a proliferative epithelial-like state (E-bCSCs), characterized by a high aldehyde dehydrogenase activity, and a quiescent, mesenchymal-like, invasive state (M-bCSCs), characterized by CD44+CD24− expression (35). This switching from E- to the M-state closely mimics the EMT program, which is associated with CSC properties and drug resistance. This observation strongly proposes that distinct bCSCs coexist within the same tumor, and thus novel combinatorial approaches targeting both CSC phenotypic states are essential to eliminate different types of bCSCs within the same tumor to reverse drug resistance phenomena. In trastuzumab-resistant HER2+ BC, combinatorial targeting of both HER2 (with trastuzumab) and IL-6 receptor (with tocilizumab) synergistically interferes with the tumor progression and metastasis by eradicating both E- and M-bCSCs (36); whereas, in the TNBC scenario, no such approach is available so far.

### How the bCSCs Originate Within a Tumor (Clonal Versus Stem Cell Model)

**Figure 2:**
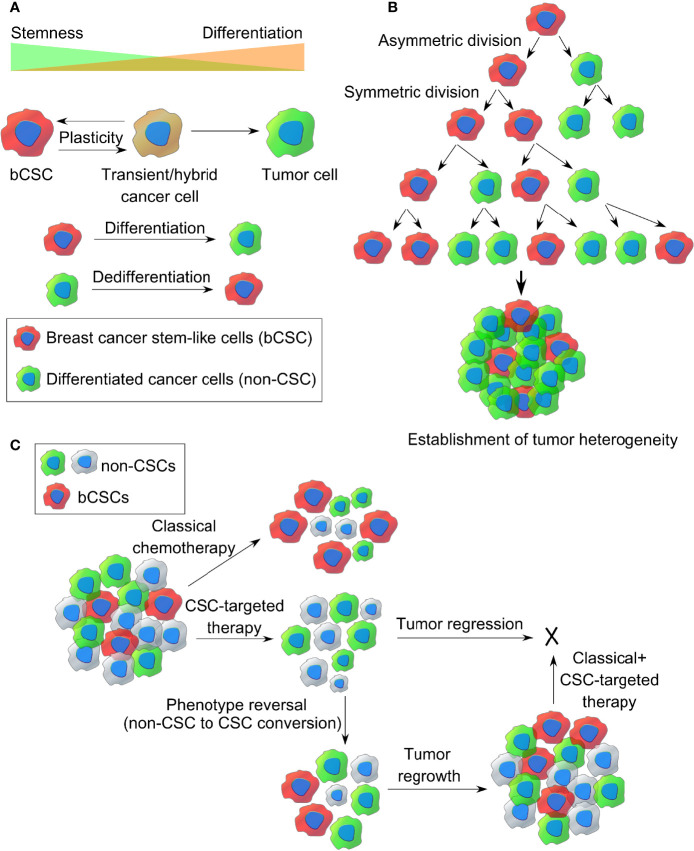
The origin of breast CSCs within a tumor. **(A)** Dynamic cancer stem cell (CSC) model of cancer cell plasticity showing switching between CSC-like state and differentiated cancer cell states (non-CSCs) through differentiation and dedifferentiation pathways. **(B)** Establishment of intratumor heterogeneity in breast cancer, resulting from symmetric and asymmetric cell divisions of breast CSCs. **(C)** Representative images of classical chemotherapy, CSC-targeted therapy, phenotype reversal, and combination therapy for target killing of breast cancer stem-like cells from TME.

There has been a great deal of debate on how CSCs originate. Clonal evolution theory and the cancer stem cell theory are the two most popular theories that shed light on the origin of this CSCs. Apart from this, CSCs are thought to be one of the determining factors establishing intratumoral heterogeneity, and both clonal evolution theory and stem cell model account for the same (37). The clonal evolution model holds an example of a nonhierarchical model where individual tumor cells are thought to undergo stochastic genetic/epigenetic changes as a function of time and serves as the platform for adaptation and selection of the fittest clones (38). Thus, each cell gets the chance to become tumorigenic or drug resistant if it accumulates enough episodes of genetic/epigenetic modifications. These changes contribute to intratumoral heterogeneity as a result of natural selection and evolution of bCSCs with better survival fitness, where those clones will expand and survive, out-compete the other nontumorigenic clones with less fitness, eventually making them extinct. This landmark theory was proposed by Peter Nowell in 1976 (39). Furthermore, these clones may change spatially and temporally and develop into a complex subclonal architecture, contributing to tumor heterogeneity. However, the dynamic CSC model represents a hierarchical model, which holds that only the CSC-like cells can develop a tumor, based on their infinite self-renewal and tumorigenic properties (refer to **Figure 2A**). According to this model, a CSC-like cell can either symmetrically divide giving rise to two new CSCs or can asymmetrically divide into a differentiated daughter cancer cell and a CSC (refer to **Figure 2B**). Hence, CSCs contribute to intratumoral heterogeneity through a differentiation program generating a range of distinct cell types within a tumor. However, this differentiation hierarchy is not only a one-way route but can also be reversible or plastic where the terminally differentiated pool of cancer cells can reverse their phenotype and acquire CSC-like properties through a dedifferentiation program, termed as “phenotype reversal”. Recent studies also indicate that different subpopulations of CSCs with varying biochemical, biophysical, and metabolic signatures may exist within a tumor, contributing to tumor heterogeneity, varied dissemination, and drug resistance potential (40). Treatment with the available chemotherapeutic drugs can kill the nonstem-like tumor cells while sparing the drug-resistant bCSCs, allowing them to survive, which eventually repopulate and develop into a tumor, leading to distant metastasis (refer **Figure 2C**). Therefore, treating a hierarchical tumor with some therapeutic agents that can specifically target and eradicate bCSCs can be the only option to get rid of CSCs and tumor recurrence. However, even if the CSC fraction is eliminated out of the TME, the remaining tumor cells may undergo phenotype reversal to replenish the CSC-like population and lead to tumor regrowth (10). Moreover, a failed radiotherapy can stimulate the transition of dormant CSCs into the “awakened state”, whereby they can enter the cell cycle and start proliferating (41).

### Characterization of bCSCs

Since bCSCs are phenotypically different from the rest of the cells present within TME, bCSCs can be identified and sorted based on some classical bCSC-specific markers like CD44, CD133, aldehyde dehydrogenase 1 (ALDH1) activity, epithelial cell adhesion molecule (EpCAM), CXCR4, ABCG2, CD34, CD49f, CD90, CD61, and breast cancer resistance protein (BRCP). However, due to the low specificity of these markers, a combination of CSC-like markers is frequently used. However, the combination of a high CD44/CD24 ratio and ALDH1+ is considered to be the most accurate and consistent way of defining bCSCs (42).

#### CD44

CD44 is a cell-surface hyaluronan acid (HA) receptor that contains an HA-binding site in its extracellular domain. Notably, HA is the major component of ECM. Hence, the CD44-HA interaction not only contributes to the cell adhesion to ECM components but also to tyrosine phosphorylation of cytoskeletal proteins, activation of RhoA/RhoC, Rac1, and Cdc42, fueling invasion and metastasis (43). Activation of these signaling pathways is essential for actin cytoskeletal remodeling, actin filament assembly, tumor cell migration, and invasion. CD44 is overexpressed in bCSCs and interestingly involves the Src kinase family proteins to initiate BC progression *via* Twist signaling (44). Moreover, CD44 contributes to chemoresistance since it upregulates the expression of multidrug resistance receptors by activating Nanog (45).

#### CD133

CD133 is another bCSC-specific marker found to be enriched in basal-like, HER2+, luminal, and TNBC subtypes. CD133-high bCSCs have been documented in tumor cell proliferation, vasculogenic mimicry, invasion, metastasis, and drug resistance (6). Croker et al. identified CD44+CD133+ALDHhigh bCSC-like cells as crucial mediators of BC metastasis (46). BRCA1-associated murine breast tumors consist of CD44+/CD24− and CD133+ cells with bCSC-like features, showing a greater intrinsic colony-forming potential that can regenerate breast tumors in NOD/SCID mice (47). Interestingly, CD133-high bCSCs augments endocrine resistance in metastatic BC *via* the IL-6/Notch signaling (48). Xenograft initiating CD44+CD49fhighCD133/2 high cells display self-renewal *in vivo* and greater tumorigenicity in ER− breast cancer (49).

#### ALDH1

Aldehyde dehydrogenase 1 is a member of NAD(P)+-dependent cytosolic isoenzymes, which is critically responsible for the oxidation of retinol to retinoic acid, required for early differentiation of stem cells. ALDH1 is a general marker of both human normal mammary stem cells and malignant mammary stem-like cells, and high ALDH1 activity is an independent predictor of poor clinical outcome and survival of BC patients (50). Interestingly, only a fraction of CD44+CD24−/low bCSC BC cells are ALDH1+ and display the highest tumorigenic potential, when compared with ALDH1− population (50). Moreover, ALDHhighCD44+CD24− and ALDHhighCD44+CD133+ bCSCs, isolated from MDA-MB-231 and MDA-MB-468 cell lines, respectively, demonstrated enhanced growth, adhesion, migration, colony formation, and invasion profile, compared with ALDHlowCD44−/low cells (46). Therefore, inhibition of ALDH activity can effectively reverse doxorubicin/paclitaxel resistance of ALDHhighCD44+ human bCSCs (51). ALDH+ bCSCs with an increased expression of interleukin-1 receptor (IL1R1) are enriched following antiestrogen therapy and held responsible for the treatment failure (52). Hence, targeting the ALDH+IL1R1+ bCSCs is crucial to reverse the drug resistance exerted by antiestrogens.

#### EpCAM

EpCAM is a glycosylated type 1 glycoprotein, expressed by human epithelial cells, and functions as an oncogenic signal transducer (53). Al-Hajj et al. showed that EpCAM+CD44+CD24−/lowLineage− fraction had a >10-fold higher frequency of tumor-initiating cells compared withto EpCAM−CD44+CD24−/lowLineage− fraction (4). Interestingly, EpCAM overexpressing BC cells can withstand greater radiation stress compared with EpCAMlow cells (54). Hence, EpCAMhigh BC cells retain the ability to form a higher number of mammospheres. Activation of the AKT pathway is also observed in EpCAM overexpressing ZR-75-1 breast cancer cell line, compared with parental cell line. Moreover, EpCAM overexpression also reflects a higher percentage of cells with an E/M hybrid state, encouraging EMT, invasion, and metastasis. EpCAM+ circulating tumor cells isolated from primary human luminal BC patients’ blood contain metastasis-initiating cells, leading to bone, lung, and liver metastasis in mice (55). Since survivin has a crucial role in bCSC chemoresistance, EpCAM aptamer-mediated survivin silencing can sensitize bCSCs to doxorubicin and reverse chemoresistance (56).

#### CXCR4

The chemokine receptor C-X-C chemokine receptor type 4 (CXCR4) is considered to be a prognostic marker of bCSCs. The metastatic cascade is initiated *via* a series of sequential steps that include local invasion and intravasation (transendothelial migration) of cancer cells from the primary tumor site into the circulation, followed by extravasation at distant sites and subsequent organ colonization (homing) (57). Cancer cells at the growing front of the tumor undergo EMT, which degrades the underlying basement membrane and ECM before intravasation. The CXCR4 receptor and its ligand, CXCL12 (SDF-1) play an important role in the dissemination of BC cells from the primary site, transendothelial migration, and eventually trafficking and homing of bCSCs. Chemokines are 8–12-kDa chemoattractant cytokines that contribute to differentiation, cell activation, and trafficking. Notably, the chemoattractant CXCL12 provides directional guidance to CXCR4+ bCSCs toward the secondary metastatic site and initiates metastasis (58–60). Hence, targeting the CXCR4-CXCL12 signaling axis could serve as an alternative approach to restrict bCSC-driven drug resistance. Additionally, CXCR4+ bCSCs show a higher vimentin/E-cadherin ratio, indicating EMT. Interestingly, CXCR4 inhibition can enhance the infiltration of cytotoxic T-cell lymphocytes (CTLs) and improve the responses to immune checkpoint blockers in metastatic BC (61). A quantitative phosphoproteomic study by Yang et al. validated the importance of CXCR4-SDF1 signaling in bCSCs and also identified several important signaling pathways in bCSCs, downstream of CXCR4-SDF1 (62).

### Evidence on Therapy-Induced bCSC Enrichment and Drug Resistance

Several studies indicate evidence on bCSC enrichment postanticancer therapy, although the underlying molecular mechanisms leading to bCSC enrichment are largely unknown. Since radiation treatment preferentially kills actively proliferating non-CSCs, there is a natural enrichment of bCSCs posttherapy. Furthermore, radiation can induce reversible transformation between CSC and non-CSC phenotype such that more CSC-like cells, with an increased level of stemness and tumorigenic potential, are generated from both normal stem cells as well as neoplastic nonstem-like cells, which ultimately leads to an increase in the absolute number of bCSCs within TME (41, 63, 64). It is hypothesized that in advanced cancer cases, the majority of the CSCs remain “dormant”, thus remaining unaffected by radiotherapy. Moreover, it is the unique potential of CSCs that can modify divisional dynamics, favoring symmetrical division, generating two identical CD44+CD24−/low daughter cells with higher radioresistance, postradiotherapy (65, 66). The number of tumor-initiating bCSCs also increases along with Notch upregulation, following a brief period of fractionated irradiation (66). Moreover, a study on non-CSCs isolated from the BC patients indicates that ionizing radiation (IR) reprograms the phenotype of differentiated BC cells and converts them into induced bCSC (i-bCSC). These i-bCSCs reflect a greater tumorigenic and mammosphere formation potential, along with a higher expression of stemness-related genes, OCT-4, Sox2, Nanog, and KIf4 (67). Furthermore, in response to IR, non-CSCs undergo radiation-induced EMT and show an increased migratory potential leading to metastasis and disease relapse, thus closely mimicking the CSC-like phenotype (68). Altogether, this evidence strongly suggests that acquisition of CSC phenotype by differentiated BC cells is an example of a direct effect of anticancer therapy, rather than a random event. Another study on glioma and breast cancer suggests that around one-third of the CSCs remain in dormancy and do not enter the cell cycle until challenged with IR (69). This refers to a mechanism whereby more “awakened CSCs” are generated from “dormant CSCs”. Moreover, radiotherapy favors oncogenic metabolism in CSCs upon their conversion from a slow-cycling “dormant” to “awakened” state, which increases their therapeutic resistance (68). Chemotherapy treatment also enhances the percentage of CD44+CD24−/low BC cells, indicative of innate chemoresistance exerted by bCSCs (70). Hence, despite eradicating the CSCs from the tumor, anticancer therapies including chemo- and radiation therapy rather help the dormant bCSCs to survive by increasing their intrinsic resistance and finally leading to tumor recurrence. Recently, an intricate link between the dormant disseminated tumor cells (DTCs) and therapeutic resistance has been documented, in the course of metastasis (71). DTCs also spread *via* the metastatic cascade and enter the blood or lymphatic circulation (57). During circulation, the DTCs undergo a reversible mitotic arrest program, followed by a long period of dormancy, termed as “quiescence”, when they remain viable but do not increase in number (72). It is hypothesized that a percentage of breast DTCs are indeed bCSCs with long half-lives (73), capable of evading immune surveillance through expression of PD-L1 and show innately higher resistance toward standard radiation, chemo-, and even immunotherapy (74). Upon reaching the distant organs, DTCs infiltrate into the local tissue stroma, although they cannot form micrometastases until dormancy is over. This period is termed as “metastatic cancer dormancy” which reflects the period between the initial therapy and disease relapse. Once the dormant DTCs get adjusted to the new microenvironment, they “awaken” from their dormant state, gain the ability to re-enter the cell cycle, and proliferate, ensuing the metastatic outgrowth (72). Interestingly, ~62% of all deaths from BC happen after 5-year survival mark, emphasizing the contribution of dormant DTCs in disease recurrence (75). Several molecular targets, including integrin α5β1, β1, α2, αvβ3, FAK, PKC, STAT3, and Cox1/2 have been identified to DTCs’ reawakening program, for which specific therapeutic agents are designed (72). Therefore, if the bCSCs could be targeted before they awaken from dormancy, metastatic dissemination and drug resistance can be potentially restricted.

### Factors Contributing to bCSC Drug Resistance Against Conventional Therapeutic Drugs

#### Vasculogenic Mimicry

**Figure 3:**
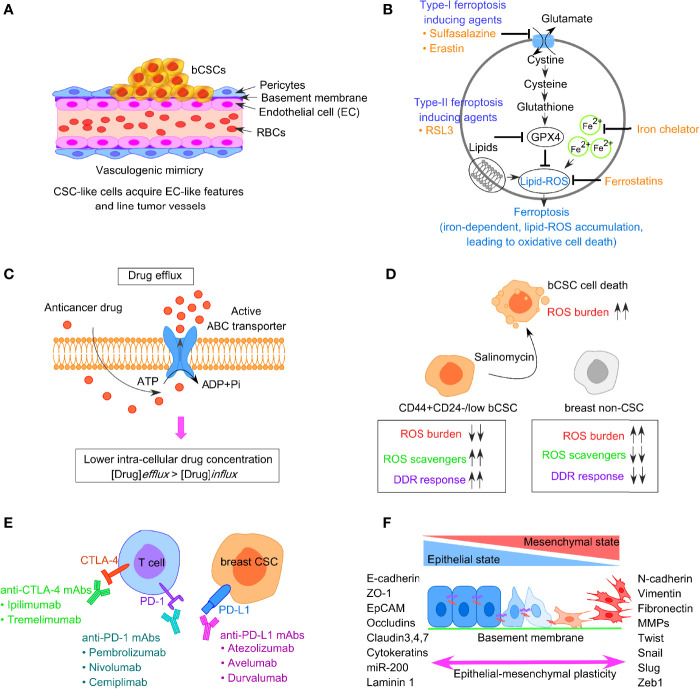
Factors responsible for bCSC-mediated drug resistance against traditional anticancer therapeutics. **(A)** Concept of vasculogenic mimicry observed in breast CSCs leading to drug resistance. **(B)** Representative image of ferroptosis pathway involving generation of lipid-ROS in an iron-dependent manner, leading to oxidative cell death of tumor cells. **(C)** Increased drug efflux due to enhanced expression of ABC transporters in bCSCs, resulting in lower intracellular chemotherapeutic drug concentration. **(D)** Low ROS burden and enhanced DNA damage repair in bCSCs. **(E)** Restoration of T-cell activity by targeting immune-checkpoint molecules like PD-1, PD-L1, and CTLA-4 to reverse CSC-mediated immune escape in breast cancer. **(F)** Epithelial-mesenchymal transition (EMT) plasticity indicating the gradual transition of cancer cells from epithelial state to mesenchymal state and the transcription factors associated with the process.

Vasculogenic mimicry (VM) is a recently defined pattern of tumor microvascularization that refers to the ability of cancer cells to organize themselves into vascular-like structures to procure nutrients and oxygen independently of normal blood vessels (76, 77). Unlike the concept of angiogenesis or vasculogenesis where the endothelial cells participate in blood vessel formation, VM particularly depends on the participation of highly aggressive tumor cells, having the endothelial phenotype, to form vessel-like structures (**Figure 3A**). VM has been reported in different types of solid aggressive tumors including BC (78–80). CD133+ breast CSCs reflect VM, with a higher expression of vascular endothelial-cadherin (VE-cadherin), along with an upregulated expression of matrix metalloproteinase, MMP-2, and MMP-9 in TNBC (81). Notably, both MMP-2 and MMP-9 are critical players in cellular plasticity and VM formation. According to Sun et al., it is the bCSCs that line the VM channels in breast tumor tissues from TNBC patients (82). Additionally, bCSCs produce more VM-related molecules like CD133 and ALDH1, to synergize VM formation (82, 83). However, cells participating in VM formation lack the classical endothelial marker CD31, and thus, administration of angiogenesis inhibitors does not affect VM formation. In this context, a phytocompound, thymoquinone (TQ), has been reported to exhibit an inhibitory effect on VM and promotes mesenchymal–epithelial transition (MET) in bCSCs derived from MDA-MB-231, in a dose-dependent manner. Moreover, CD44+CD24− bCSCs when incubated with TQ can interfere with rhodamine-123 efflux and decrease stemness. This observation indirectly denotes that thymoquinone relieves the drug-resistance properties of bCSCs (84). Mechanistically, TQ suppresses the PI3K and Wnt3a signaling, leading to the reduction of the p-Akt/Akt ratio, and has the potential to reduce the number of bCSCs.

#### Decreased Ferroptosis in bCSCs

Ferroptosis is an iron-dependent mechanism of regulated cell death, which is characterized by the intracellular accumulation of lipid-based ROS, ultimately resulting in the loss of membrane integrity (**Figure 3B**) (85). Notably, lipid-ROS is detoxified in a GPX4-catalyzed enzymatic reaction, which uses glutathione as a reducing agent. Hence, ferroptosis can be triggered either by inhibiting GPX4 enzymatic activity or depleting glutathione. Type I ferroptosis-inducing compounds, including sulfasalazine and erastin block the amino acid transporter required for cysteine import to synthesize glutathione. Type II drugs, such as RSL3, interfere with GPX4 peroxidase activity. Mechanistically, the execution of ferroptosis requires a high concentration of intracellular iron. Ferritin, the intracellular iron-storing protein, can release iron to initiate ferroptosis. The released iron can yield lipid-ROS in an autoamplifying manner. Ferroptosis can be inhibited by the presence of iron chelators and activated by transferrin and its receptor (86). Hence, sensitizing tumor cells to ferroptosis appears as a possible therapeutic approach for BC treatment. Notably, drug-tolerant BC cells show a dependency on the GPX4 activity, thus inhibition of GPX4 can potentially overcome BC drug resistance (87). Taylor et al. reported an array of ferroptosis-inducing small molecules that can selectively kill bCSCs with the mesenchymal phenotype *in vitro* (88). TNBC cells are highly susceptible to cysteine starvation, leading to ferroptosis and necroptosis, *via* the GCN2-eIF2α-ATF4-CHAC1 pathway (89). Since cysteine serves as the substrate for glutathione synthesis to prevent ferroptosis, depleting the pool of cysteine can sensitize BC cells to ferroptosis (89). Another synthetic derivative from salinomycin, ironomycin (AM5), can trigger cell death in bCSCs, both *in vitro* and *in vivo*, by sequestering iron in lysosomes, which further indicates that iron homeostasis plays a crucial role in bCSC survival (90). A novel nanoparticle, ferritin-bound erastin, and rapamycin (NFER) has shown robust ferroptosis-inducing properties by interfering GPX4 in 4T1 orthotopic BC mouse model (91).

#### Increased Autophagy in bCSCs and Drug Resistance

Autophagy is an evolutionarily conserved self-degradation process that recycles intracellular nutrients, growth factors, and energy to sustain survival and cellular activities during stress like hypoxia, nutrient deprivation, and ischemia (92). Autophagy provides bCSCs with metabolic flexibility that becomes a prerequisite for their survival in oxygen- or nutrient-poor TME (93). Autophagy contributes to bCSC dormancy, stemness, maintenance, and drug resistance (94–96). Chaperone-mediated autophagy (CMA) and macroautophagy are two different modes of the autophagy process, documented in mammalian cells. Autophagy can elevate bCSC number, and thus develop drug resistance to conventional chemotherapies (97). Furthermore, autophagy-related genes (ATGs) such as ATG4A, ATG5, ATG12, LC3-B, and Beclin1 are expressed in dormant bCSCs, promoting bCSC survival and sustaining bCSCs over the progression of BC (96). Expression of Beclin1 is noted to be higher in mammospheres derived from BC cell lines, MCF-7 and BT474, compared with the adherent cultures (98). Moreover, the expression of lysosome-associated membrane protein type 2A (LAMP2A), involved in the CMA pathway, is augmented in the course of BC metastasis (99). Autophagy of cancer-associated fibroblasts (CAFs) also contributes to TNBC proliferation and progression. Notably, autophagy-relevant Beclin1 and LC3-II/I protein conversion levels are higher in CAFs, compared with the normal fibroblasts in TNBC (100). Since autophagy serves as one of the factors in malignant growth, inhibition of autophagy can suppress tumor growth. Pharmacological targeting of the autophagic flux with salinomycin can reduce bCSC-driven drug resistance, interfere with their stemness, and also compromise the bCSC tumorigenic potential (101).

#### Enhanced Drug-Efflux in bCSCs

CSCs often express a higher level of ATP-binding cassette (ABC) transporters that facilitate them to survive chemotherapy, aiding in the survival of drug-resistant CSCs (102). ABC transporters can efficiently expel chemo drugs like anthracycline or taxanes out of the cells and can eventually lead to the acquisition of drug resistance phenotype in bCSCs (**Figure 3C**). This group of proteins is located on the cell membrane and thus can allow transmembrane transportation of different toxic molecules. Notably, CSCs exhibit a higher expression of ABC transporters, such as ABCB1 (MDR1), ABCG2 (BCRP1), ABCC11 (MRP8), and ABCB, which are positively correlated with CSC-mediated drug resistance (103, 104). The activity of drug efflux proteins can be monitored through the transport of fluorescent dyes like rhodamine and Hoechst 33342 (105). Based on this property, the CSCs can be isolated from non-CSCs by fluorescent-activated cell sorter (FACS). This fraction of cells termed as “side population” (SP), is identified as CSCs since these cells fall along the side of the cellular distribution on the FACS profile. Downregulating ABCG2 with wedelolactone-encapsulated PLGA nanoparticles increases the chemosensitivity of bCSCs (106). The Sox2-ABCG2-TWIST1 axis contributes to chemoresistance and stemness in TNBC, indicating the importance of ABCG2 as a potential bCSC-specific target in TNBC patients (107). Moreover, simultaneous blocking of ABCG2 and antiapoptotic gene BCL2 with SiRNA in bCSCs leads to better chemotherapeutic response to doxorubicin (108). Dofequidar, an ABC transporter inhibitor, increases the chemosensitivity of bCSCs in advanced or recurrent BC patients, when administered in combination with chemo drugs like doxorubicin, fluorouracil, and cyclophosphamide (109). ABC transporters not only participate in establishing the drug resistance *via* increased efflux of chemo drugs but also contribute to EMT (110). ABCB1 is another group of ABC transporter implicated in the chemoresistant nature of CSCs and induction of EMT (111, 112). Therefore, the combined application of chemotherapeutic drugs and ABC inhibitors should be employed to kill the bCSCs (111).

#### Enhanced DNA Repair in bCSCs

Although cancer cells show a reduced DNA damage repair (DDR) mechanism and reflect many mutations and genomic instability, CSC-like cells exhibit a highly dynamic DDR system that protects the DNA effectively (113). Both chemotherapy drugs and radiotherapy can induce DNA damage. Mechanistically, radiotherapy contributes to DNA damage through the production of water-derived free radicals and ROS that avidly interacts with DNA, protein, and lipids. The generation of ROS, in turn, switches on the DDR pathway. However, in contrast to non-CSCs, CSCs have a low ROS burden and augmented DNA repair system (refer to **Figure 3D**). An increased level of ROS scavengers in CSCs maintains low levels of ROS that protect the CSCs from ROS-mediated DNA damage and apoptosis. The ROS scavenger, N-acetylcysteine, can restore both CSC and EMT phenotypes (114). A novel compound, salinomycin, can effectively target the CSC niche and kill CD44highCD24−/low bCSCs as it upregulates the ROS levels (115). The radioresistance property of CSC-like cells is linked with cell-cycle kinetics, which is reflected by a significant increase in the doubling time and Chk1/Chk2 basal activation level (116). The elongated cell-cycle window, therefore, offers more time to repair the genetic defects in CSCs. When the DNA defects are corrected, CSCs again enter into the cell cycle from the quiescent state and escape apoptosis. Therefore, targeting DDR could reverse therapeutic resistance. Frizzled 5 (FZD5), a member of the FZD family, contributes to DDR, G1/S transition, proliferation, stemness, and chemoresistance in TNBC (117). FZD5 knockdown suppresses the expression of CD133, ALDH, EpCAM, and Oct-4, thus potentially overcoming chemoresistance and recurrence in TNBC. Likewise, CCR5 directs the DDR mechanism and bCSC expansion (118). CCR5 antagonists, vicriviroc and maraviroc, can substantially increase cell death caused by DNA-damaging chemo drugs. MYC and MCL1 also cooperatively function in bCSC maintenance in TNBC patients *via* increasing ROS production and HIF-1α expression (119).

#### Immune Escape in bCSCs and Drug Resistance

CSCs are a crucial driver in immune evasion, metastasis, and drug resistance. Substantial evidence suggests a reciprocal interaction between CSCs and immune cells, and that CSCs adopt different strategies to circumvent immune attacks mediated by different immune cell types within TME. This, in turn, contributes to CSC expansion and mediate protumorigenic immune function, leading to CSC-specific avoidance of immune detection and destruction of immune cells. Tumor-associated macrophages (TAMs), tumor-associated neutrophils (TANs), myeloid-derived suppressor cells (MDSCs), DCs, tumor-infiltrating lymphocytes (TILs), B cells, natural killer (NK) cells, and T regulatory (Treg) cells, and proinflammatory cytokines secreted by these cells, are therefore crucial for maintaining an immune-resistant phenotype. Treatment of bCSCs with conditioned medium from TAMs results in an upregulated expression of Oct3/4, Sox2, Nanog, and ALDH1 activity (120). MDSCs also lead to the enrichment of bCSCs *via* IL-6/STAT3 and NO/Notch signaling, leading to the suppression of T-cell activation (121). The T-cell inhibitory molecule, PD-L1 is overexpressed on the bCSC cell surface, compared with their differentiated counterparts, and is dependent on PI3K/AKT and Notch signaling pathway (122). Interestingly, PD-L1 expression is upregulated in response to EMT induction and facilitates the immune escape of bCSCs (123, 124). Moreover, bCSCs are not only resistant to chemotherapy but also immunotherapy (125). Therefore, restoring the T-cell activity by manipulating immune checkpoint molecules, targeting either PD-1/PD-L1 (nivolumab/pembrolizumab) or CTLA-4 (ipilimumab) can be an effective strategy (**Figure 3E**). Also, immunosuppressive cytokines secreted by breast CSCs (IL-4, IL-6, IL-8, IL-10, and IL-13) result in therapeutic resistance, increased EMT, metastasis, and recruitment of immunosuppressive immune cell types like Treg and MDSCs (126). A high circulating level of IL-6 contributes to disease recurrence, tamoxifen resistance in luminal BC, and trastuzumab resistance in HER-2 enriched BC. Targeting IL-6 receptor with monoclonal antibody tocilizumab, hence, suppresses metastatic potential of bCSCs and enhances the cytotoxicity of cisplatin against TNBC (127).

#### bCSC-Driven EMT, Metastasis, and Drug Resistance

Like normal tissue stem cells, EMT and the reverse process MET are critical to CSC features. Substantial evidence exists that correlates EMT plasticity to the emergence of dedifferentiated cells with CSC phenotype, ultimately driving metastasis and drug resistance (128, 129). EMT inducers such as TGF-β and receptor tyrosine kinase (RTK) ligands modulate gene expression patterns through complex signaling networks (112). This results in the upregulation of transcriptional repressors like Snail, Slug, Zeb1/2, Twist, and E47. This group of proteins, then, interacts with the promoter sequence of adherens junction protein, E-cadherin, recruits histone deacetylases (HDACs), and induces its chromatin condensation, leading to transcriptional repression of E-cadherin. EMT involves the dissolution of cell–cell adherens junction barriers, loss of apico-basolateral polarity of epithelial cells, along with increased expression of mesenchymal markers such as fibronectin and vimentin (**Figure 3F**) (128). This, in turn, aids in gaining motile characteristics of cancer cells post-EMT. Mani et al. indicated that when the EMT program is induced in immortalized human mammary epithelial cells through ectopic expression of Twist, Snail, or TGF-β treatment, the cells exhibited mesenchymal appearances, developed many stem-like properties, and had the potential to form mammary tumors in mice (128). Importantly, the metastatic cancer cells with mesenchymal-like features generated post-EMT, exhibited CD44high/CD24low signature, and formed mammospheres whereas CD44low/CD24high cells could not. Al-Hajj et al. reported that disseminated BC cells found in pleural effusions are enriched in CD44highCD24−/low bCSCs (4). Interestingly, the EMT-associated emergence of bCSCs is induced by CD8+T cells that stimulate dedifferentiation of BC cells into bCSCs (130). An increased expression of stroma cell-related genes, attributed to the EMT program, could be linked to drug resistance in BC (131). Hence, blocking the EMT program can eventually interfere with CSC maintenance and innate or acquired drug resistance (70). Therapeutic intervention of micro-RNAs can provide an additional strategy to disrupt the EMT-CSC deadly axis. Application of HDAC inhibitors and “differentiation-inducing” agents are also believed to fetch clinical benefits to BC patients.

## Mechanism of bCSC-Mediated Drug Resistance to Cancer Therapy

### Resistance to Chemotherapy

Chemotherapy involves chemo drugs given to the BC patients either intravenously or orally, along with other treatments like surgery, radiation, or hormonal therapy. Through the bloodstream, the chemo drugs reach the cancer cells and kill them. Adjuvant and neoadjuvant chemotherapies are two different modes of chemotherapies. In adjuvant chemotherapy (following surgery), the chemo drug is given to kill the cancer cells that have been left behind or could not be seen in imaging tests; whereas, in neoadjuvant chemotherapy, the chemo drug is given to shrink the breast tumor to reduce the requirement of extensive surgery. However, several lines of evidence indicate bCSC enrichment following exposure to chemo drugs, resulting in multidrug resistance (MDR). Here, we focus on the variety of chemo drugs (refer to **Table 1**), their mode of action, and potential mechanisms of chemoresistance exerted by bCSCs.

**Table 1:** Different chemotherapeutic modalities in clinical practice and novel therapeutic drugs being developed against BC subtypes and their mechanism of action.

Breast cancer subtype	Drug	Biological target (mechanism of action)
**Hormone positive**	**In clinical practice**	
	Tamoxifen	Competitively inhibits interaction between ER and estrogen
	Fulvestrant	SERD, competitively inhibits estrogen to occupy ER, ER degradation
	Aromatase inhibitors (AIs) (exemestane, anastrozole, letrozole)	Blocks conversion of androgens to estrogens
	Leuprolide	Reduces production of estrogen and progesterone by the ovary by blocking effects of GnRH on the pituitary gland
	Goserelin	Luteinizing hormone-releasing hormone (LHRH) agonist, stops LH production, blocks release of estrogen
	Palbociclib (FDA approval: February 2015)	CDK4/6 inhibitors for advanced stage BC along with letrozole
	Ribociclib (FDA approval: March 2017)	CDK4/6 inhibitors for advanced stage BC along with letrozole
	Abemaciclib or verzenio (FDA approval: October 2021)	CDK4/6 inhibitors for treatment of early-stage BC
	Everolimus (FDA approval: July 2012)	mTOR inhibitor, sensitizes hormone-receptor-positive BC to exemestane
	**In pipeline**	
	Buparlisib (BKM120)	Pan-class I PI3K inhibitor, combination therapy with fulvestrant, phase III trial (NCT01610284)
	Alpelisib	PI3K inhibitor, inhibiting p110 alpha; combination therapy with fulvestrant, phase III trial (NCT02437318)
	Taselisib	Alpha-specific PI3K inhibitor; combination therapy with fulvestrant, phase III trial (NCT02340221)
	Entinostat	HDAC inhibitor, phase II trial with exemestane (NCT02115282)
	Vorinostat	HDAC inhibitor, in combination with tamoxifen, terminated (NCT01194427)
	Irosustat	Steroid sulfatase inhibitor with AI, phase II trial completed (NCT01785992)
**HER2 enriched**	**In clinical practice**	
	Trastuzumab	Anti-HER2 mAb interacting with extracellular domain IV of HER2
	Pertuzumab	Anti-HER2 mAb targeting HER2 extracellular domain II, inhibiting HER2 heterodimerization with EGFR, HER3, and HER4
	Lapatinib	Tyrosine kinase inhibitor (TKI) targeting both EGFR and HER2, interacts at ATP-binding site of kinases
	Ado-trastuzumab emtansine	Anti-HER2 mAb conjugated with microtubule inhibitor emtansine
	Margetuximab (FDA approval: December 2020)	HER2-targeted antibody for metastatic HER2+ BC
	Tucatinib (FDA approval: April 2020)	HER2 inhibitor, used in combination with trastuzumab and capecitabine (Xeloda) in metastatic HER2+ BC
	**In pipeline**	
	Patritumab	Anti-HER3 mAb in combination with trastuzumab and paclitaxel in phase I/II trial completed (NCT01276041)
	Buparlisib with lapatinib and pilaralisib with trastuzumab	Pan class-I PI3K inhibitors, phase I/II trial (NCT01589861), phase I/II trial (NCT01042925)
	Lonafarnib	Inhibits Ras activity, combination therapy with trastuzumab and paclitaxel, phase I completed (NCT00068757)
	NeuVax + trastuzumab	Immunotherapy for treatment of early-stage HER2+ BC; phase IIb trial (NCT02297698)
	Ridaforolimus with trastuzumab	mTOR inhibitors, phase II trial completed (NCT00736970)
	Sirolimus with trastuzumab	mTOR inhibitors, phase II trial completed (NCT00411788)
	MK-2206	Allosteric pan-Akt inhibitor; combination therapy with trastuzumab and lapatinib, terminated (NCT00963547)
**Triple-negative**	**In clinical practice**	
	Anthracyclines	Topoisomerase II inhibitors, stabilize DNA breaks and ensuing tumor cell death
	Taxanes	Microtubule-stabilizing agent, stabilize GDP-bound tubulin in microtubule, G2/M arrest, cell death
	Olaparib	PARP inhibitor, blocks repair of single-strand DNA breaks by base excision repair (BER) system
	Talazoparib	PARP inhibitor
	Bevacizumab	Antiangiogenic mAb against VEGF bevacizumab + docetaxel anti-VEGF mAb
	Atezolizumab (FDA approval: March 2019)	Anti PD-L1 antibody as first-line therapy to locally advanced or metastatic PD-L1-positive TNBC patients
	Pembrolizumab (FDA approval: October 2021)	Anti PD-1 antibody for high-risk early-stage TNBC
	Trodelvy (sacituzumab) (FDA approval: 2020)	Trop-2 directed antibody and topoisomerase inhibitor drug conjugate for metastatic TNBC patients
	**In pipeline**	
	Cetuximab + cisplatin or carboplatin	Anti-EGFR mAb for metastatic TNBC, phase II completed (NCT00463788)
	Glembatumumab vedotin	mAb-cytotoxic drug conjugate targeting glycoprotein NMB in TNBC, phase II completed (NCT01997333)
	Dasatinib + cetuximab + cisplatin	Src inhibitors, tested in TNBC cell lines

The clinical trial number for the drugs in pipeline has been mentioned according to ClinicalTrials.gov.

#### Paclitaxel Resistance

**Figure 4:**
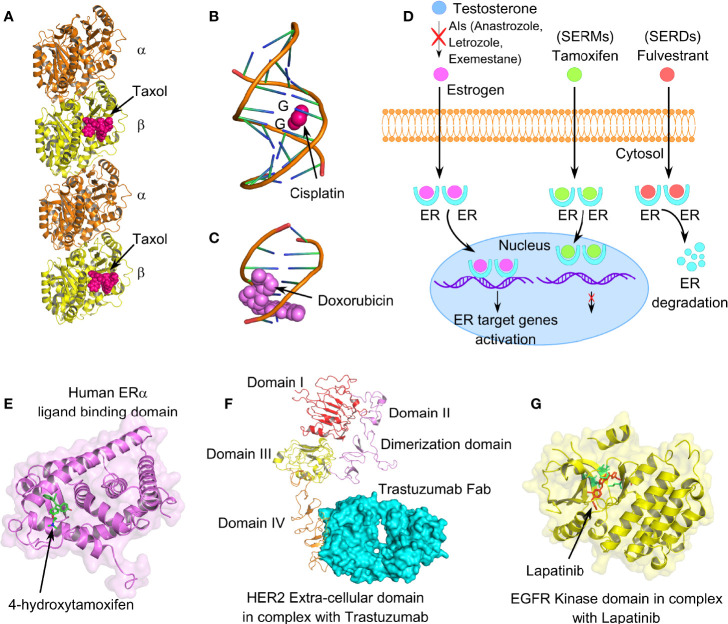
Mechanism of action of different anticancer drugs for the treatment of breast cancer. **(A)** Tubulin dimers stabilized with microtubule-stabilizing drug paclitaxel (PDB code: 6WVR). **(B)** Interaction of chemo drug cisplatin with double-stranded DNA (PDB code: 1AIO), forming major adducts of cisplatin with guanine nucleotides. **(C)** Doxorubicin intercalation with DNA base pairs (PDB code: 2DES). **(D)** Cartoon representation of mechanism of action of endocrine therapeutic drugs, such as selective estrogen receptor modulators (SERMs), selective estrogen receptor degrader (SERDs), and aromatase inhibitors (AIs). **(E)** Human ER-α-ligand-binding domain in complex with tamoxifen (PDB code: 3ERT). **(F)** Extracellular domain IV of HER2 in association with recombinant humanized IgG1 monoclonal antibody, trastuzumab (PDB code: 6OGE). **(G)** EGFR kinase domain in complex with lapatinib, a selective receptor tyrosine kinase inhibitor, targeting both EGFR and HER2 (PDB code: 1XKK). Lapatinib interacts in the ATP-binding pocket of EGFR (L718, V726, A743, M793, and L844); highlighted in lemon green.

Paclitaxel, a first-line therapeutic agent for the treatment of metastatic BC, is a microtubule-stabilizing agent. It interferes with microtubule dynamic instability at nanomolar concentrations, thus leading to G2/M mitotic arrest and apoptosis in BC cells (**Figure 4A**). Paclitaxel resistance seems to be one of the primary obstacles that lead to chemotherapeutic failure in BC. HER2/β-catenin pathway mediates paclitaxel resistance in BC cells, and hence suppression of the HER2/β-catenin signaling can overcome paclitaxel resistance (132). Penfluridol (PFL) treatments that suppress both HER2/β-catenin pathways significantly inhibit the survival of paclitaxel-resistant BC cells. Notably, paclitaxel resistance increases both CD44+CD24− bCSC content and sphere-forming ability in the paclitaxel-resistant SUM159 metastatic TNBC cell line (133). According to this report, dasatinib, an Src family kinase inhibitor, induces epithelial differentiation of mesenchymal TNBC cells and sensitizes TNBC cells to paclitaxel therapy through targeting bCSCs. An increased association of ALDH1 expression has been noted in paclitaxel-resistant BC patients (134). TNBC cell lines, such as MDA-MB-231, SUM-149, and SUM-159 show an enhanced activity of hypoxia-inducible factors (HIFs) and their target gene products, with chronic exposure to paclitaxel therapy. Furthermore, chemotherapy-induced HIF activation results in bCSC enrichment through IL-6 and IL-8 signaling and enhanced expression of MDR-1 (135). Hence, combinatorial therapies including HIF inhibitors along with paclitaxel chemotherapy are being tested in clinical trials to explore its efficacy in BC patients.

#### Platinum Resistance

Platinum-based chemotherapeutic drugs, such as cisplatin, oxaliplatin, carboplatin, nedaplatin, and lobaplatin are frequently the choice of drugs for treating advanced BC cases, including TNBC (136, 137). Mechanistically, platinum-based drugs interact with guanine and adenine nucleotides, forming platinum-DNA nonfunctional adducts, disrupting DNA double-helical structure, and eventually inhibiting cell division (**Figure 4B**) (138). However, drug resistance associated with platinum therapy and the numerous side effects that it causes have been a long-standing concern for BC patients. According to Sledge et al., only 47% of the BC patients with metastatic BC are partially sensitive to platinum therapy (139). Several lines of evidence indicate a crucial contribution of bCSCs in developing and maintenance of platinum resistance. In this context, a novel drug disulfiram (DSF) can reverse cisplatin resistance in different BC cell lines through inhibiting ALDH enzymatic activity and interfering with the expression of Oct4, Sox2, and Nanog in bCSCs. IL-6 secreted by breast tumor-derived mesenchymal stem-like cells (MSCs), augments cisplatin resistance *via* STAT3 signaling (140). Although neutralizing IL-6 can partially interfere with the IL6-STAT3 axis and reverse the cisplatin resistance, the specific role of bCSCs remains unclear. Notably, there is the active involvement of PI3K/AKT/NF-ĸB signaling in enrichment as well as maintenance of breast CSCs. Cisplatin is known to stimulate transcriptional upregulation of PI3KCA, thereby triggering PI3K/AKT signaling in platinum-resistant cells. However, a recent report emphasizes that mechanistically cisplatin leads to CSC enrichment in platinum-resistant cells through the NF-ĸB-TNF-α-PI3KCA loop (141).

#### Anthracycline Resistance

Anthracyclines, antibiotics extracted from *Streptomyces* bacteria, administered as broad-spectrum chemotherapeutic drugs in BC patients, are topoisomerase II inhibitors (**Figure 4C**) (142). Since, topoisomerases modify DNA topology by breaking or rejoining DNA double strands, inhibiting its catalytic activity stabilizes DNA breaks, eventually causing cell death. This class of drugs includes doxorubicin, daunorubicin, and epirubicin. Resistance to anthracyclines has been linked with multiple factors, such as the acquisition of MDR due to overexpression of drug efflux pumps and permeability glycoprotein-1, alteration of topoisomerase II activity, CSC enrichment, altered DNA repair, and metabolic reprogramming (143). A 4-day exposure to doxorubicin and paclitaxel, followed by a 2-day recovery, leads to significant enrichment of CD44highCD24−/low bCSCs (144). In this context, cardamonin, a small molecule, significantly prevents bCSC enrichment, when administered along with chemotherapeutic drugs, *via* downregulation of IL-6, IL-8, NF-ĸB, and STAT3 signaling (144). Knockdown of Annexin A3 also influences the drug sensitivity of bCSCs to doxorubicin *via* an upregulation of drug uptake, inhibits metastasis, and exhibits a change in heterogeneity and plasticity in bCSCs (145).

### Resistance to Endocrine Therapy

Endocrine therapy is an effective mode of treatment for the ER+ BC cases that blocks ER signaling, depriving the growing tumor of estrogen (146, 147). ER signaling plays a crucial role in BC proliferation, invasion, and angiogenesis. Mechanisms, through which the endocrine therapy works (refer to **Table 1**), can be categorized into (1) SERMs, (2) aromatase inhibitors (AIs), (3) CDK4/6 inhibitors, and (4) SERDs (**Figure 4D**) (148, 149). SERMs function by sitting in the ER of breast tissues, blocking the estrogen from interacting with the ER, and hence the cells can no longer grow and multiply (150). AIs work by blocking the function of the aromatase enzyme that converts androgen into estrogen (151). CDK4/6 inhibitors are generally used in combination with endocrine therapy to treat hormone-receptor-positive but HER-2 negative metastatic BC (152, 153). CDK4/6 is required by BC cells for cell-cycle division. BCSCs develop resistance to endocrine therapy in ER+ BC and are mainly responsible for the failure of endocrine therapy (154). Therefore, specific targeting of drug-resistant bCSCs could serve as a potential therapeutic strategy in overcoming hormonal therapy resistance.

#### Tamoxifen Resistance

ER-α-positive BC cases constitute around 70%–75% of overall BC incidence. Although, tamoxifen (TAM) has been the fundamental mode of endocrine therapy for the treatment of ER+ BC patients for the last three decades, acquired TAM resistance is frequently held accountable for the disease relapse (155). TAM competitively inhibits the interaction of estrogen ligand with ERs (**Figure 4E**). Most importantly, an increased proportion of bCSCs in advanced BC patients have a potential contribution to TAM resistance and breast tumorigenesis (156). Poorly differentiated breast tumors contain a higher percentage of CSC-like cells than well-differentiated breast tumors (157). TAM-resistant BC cells retain stem-like properties (158). Notably, TAM-resistant MCF-7 cells showed increased proliferation rate, enhanced mammosphere formation ability, increased mRNA expression of OCT-4, SOX-2, and CD133, and increased EMT signature, compared with wild-type MCF-7 cells (158). In a parallel study, Wang et al. indicated that TAM-resistant MCF-7 cells contain a higher proportion of CD44+CD24−/low bCSCs, exhibit lesser sensitivity to Adriamycin compared with wild-type MCF-7 cells, and express SOX-2 as a biomarker for TAM resistance (159). Serine phosphorylation, particularly at Serine 118, has been documented for activating the N-terminal transcriptional function of ER-α. SOX-2 can reprogram the non-genomic estrogen signaling and augment bCSC proportion through phosphorylation of ER-α at serine 118, making it hypersensitive to circulating estrogen (160). Phosphorylation, ubiquitination, and other posttranslational modifications play an important role in activating ER and its coregulators and can influence the sensitivity to different endocrine therapies (161). Therefore, inhibition of SOX-2 could restore the sensitivity of BC cells to TAM (162). Furthermore, ER splicing variants, including estrogen-related receptors (ERRs), and the recently identified estrogen receptor α-variant (ER-α36) are involved in TAM resistance and estrogen hypersensitivity (163). However, the contribution of ER-β in bCSC-mediated TAM resistance is still under investigation. Upregulation of different growth factors including HER2, epidermal growth factor receptor (EGFR), and insulin-like growth factor 1 receptor (IGF1R) has been documented in BC endocrine resistance, although direct evidence has been found in support of PI3K-mediated TAM resistance (164). Hence, targeting PI3K and IGF1R is considered a major therapeutic target to reverse TAM resistance in bCSCs. Different signaling pathways, such as Wnt and Notch, induce TAM resistance, promoting bCSC activity in TAM-resistant MCF-7 cells, while inhibition of these pathways could overcome TAM resistance (165, 166). A positive correlation has been noted between activation of Hedgehog (Hh) signaling and reduction in disease-free or recurrence-free survival in BC patients, which can even result in TAM resistance (167). The intervention of Hedgehog (Hh) signaling, thus, can potentially interfere with bCSC proliferation, migration, and invasion and reverse TAM resistance. Therefore, inhibition of Notch, Hedgehog (Hh), and Wnt/β-catenin signaling pathways should serve as another strategy to overcome TAM resistance in bCSCs.

#### Fulvestrant Resistance

Fulvestrant is a selective estrogen receptor degrader (SERD) administered in both first and subsequent lines of treatment in ER-α+ metastatic BC patients (149, 168). Fulvestrant competitively inhibits estrogen to occupy the ER, eventually promotes degradation of the receptor, and thus interferes with estrogen signaling in breast tumor tissues (169). Unfortunately, there has not been extensive research done on fulvestrant resistance in bCSCs as well as the molecular mechanisms responsible for the resistance. Dysregulation of both G protein-coupled estrogen receptor-1 (GPER) and CDK6 are associated with fulvestrant resistance in BC (170). Notably, GPER-induced signaling is essential for the survival of bCSCs (171). Very recently, Kaminska et al. reported that cyclin E2 overexpression has been recognized as a biomarker for persistent fulvestrant-resistant metastatic BC and reduced disease-free survival (172). However, AI-resistant BC cells, having a higher proportion of bCSC-like cells and increased stemness, are inhibited by fulvestrant (173). Several signaling pathways, such as MEK/ERK, NF-ĸB, EGFR, PI3K/AKT, and β-catenin have been implicated so far to fulvestrant resistance in BC. MiRNA-221/222 confers estrogen-independent growth and fulvestrant resistance in BC through multiple signaling networks. Strikingly, miR-221/222 contributes to acquired fulvestrant resistance through activation of the β-catenin pathway, and miR-221/222 has recently been documented in CD44+CD24−/low bCSCs (174).

#### Aromatase Inhibitor Resistance

AIs constitute the first-line therapeutic approach for the treatment of ER+ BC in postmenopausal women (175, 176). AIs deplete the circulating level of estrogen in the human body by interfering with estrogen biosynthesis through blocking aromatase activity (177). Hence, in the presence of AIs, estrogen production is inhibited, which slows down tumor progression in ER+ BC settings. When treating early-stage ER+ BC, AIs are frequently the choice of hormonal therapy over TAM due to the fewer side effects it causes. However, acquired AI resistance may develop in over 20% of early-stage BC patients and found to be inevitable in metastatic BC patients (178). Acquired AI resistance involves a switch from dependence on ER signaling to growth-factor-mediated signaling, such as HER2 signaling (179). Both cancer-cell intrinsic (enhanced activity of FGFR, ERBB2, and IGF1R and the downstream signaling of PI3K-AKT-mTOR and MAPK pathways) and extrinsic mechanisms (interaction of TME with other cell types) cumulatively coordinate the development and maintenance of AI resistance (180). AIs are classified into 2 subtypes—steroidal (type I) and nonsteroidal (type II). The three different AIs anastrozole (nonsteroidal), letrozole (nonsteroidal), and exemestane (steroidal) are being used in adjuvant therapy as the first line of treatment modality for both early and metastatic BC in postmenopausal women. BCSCs in ER-α+ settings reflect an activated PI3K signaling, which confers endocrine resistance including AI resistance (181). Notably, different PI3K inhibitors such as alpelisib, buparlisib, and taselisib (https://clinicaltrials.gov/ct2/show; ClinicalTrials.gov Identifier: NCT02437318, NCT01610284, and NCT02340221) are being administered as novel therapeutic drugs in phase III clinical trials for the treatment of breast cancer AI resistance (**Table 1**). The expression of HIF-1α has also been recognized as a biomarker and therapeutic target that promotes AI resistance (179).

### Resistance to Targeted Therapy

HER2 is an oncogenic RTK, which is frequently genetically amplified or overexpressed in around 15%–20% of invasive BC cases (182). However, although the emergence of anti-HER2 drugs, trastuzumab and lapatinib, significantly improved the clinical outcome in HER2-enriched BC, the associated drug resistance problem poses challenges to effective treatment. Resistance to anti-HER2 drugs occurs due to the presence of bCSCs in the tumor milieu that can remain “hidden” from the activity of these drugs (111). Therefore, we need to understand the mechanisms responsible for the associated drug resistance followed by the application of anti-HER2 drugs to encounter the involvement of bCSCs in therapeutic resistance (refer to **Table 1**).

#### Trastuzumab Resistance

Amplification of ERBB2 (HER2) is associated with clinically aggressive breast tumors, shorter disease/recurrence-free survival, and poor overall survival (183). Trastuzumab is a recombinant humanized IgG1 monoclonal antibody that interacts with extracellular domain IV of ERBB2, inhibiting dimerization between ERBB2 and other EGFR family members (**Figure 4F**) (184). Although HER2+ BC responds quite well to trastuzumab (Herceptin™) therapy plus chemotherapy in the early stages of the disease, acquired resistance, however, to trastuzumab after 1–2 years of treatment is a frequent event following metastasis (185). Factors like HER2 degradation, overexpression of other RTKs, mutation of PI3KCA (PI3K catalytic subunit p110α), and loss of Phosphatase and Tensin Homolog Deleted on Chromosome 10 (PTEN) tumor-suppressive function have been linked with trastuzumab resistance (186). Continued application of trastuzumab in HER2+ cells with loss of PTEN encourages EMT and transforms HER2+ BC to TNBC (187). Strikingly, these transformed cells frequently exhibit mesenchymal features along with mesenchymal-specific gene expression profile, although the parental HER2+ cells show epithelial morphology with epithelial-specific gene signature. Since bCSCs exhibit chemoresistance to small-molecule targeted therapy, exploring the mechanism of trastuzumab resistance must have clinical implications. BCSCs confer drug resistance by activation of different prosurvival pathways, such as PI3K/AKT, NFĸB, and JAK/STAT pathways (188). Thus, CD44+CD24−/low bCSC phenotype serves as a prognostic factor for clinical outcome and predictive factor for poor trastuzumab response in patients with HER2+ BC. Importantly, PI3K/AKT/mTOR activation has been implicated in both *de novo* and acquired trastuzumab resistance (189). Since PTEN loss and mutation of PI3KCA lead to aberrant downstream activation of PI3K/AKT/mTOR pathway, which in turn sustains bCSC population, both the factors correlate with trastuzumab resistance (190). Therefore, combining PI3K/AKT/mTOR inhibitors along with HER2 targeting drugs to overcome trastuzumab resistance provides an active area of research. Pan-class I PI3K inhibitors, such as buparlisib and pilaralisib when administered with trastuzumab (189), lapatinib (191), or trastuzumab and paclitaxel (192), are proven to be safer and successful in HER2+ advanced stage BC patients. IL-6-mediated bCSC expansion is another independent mechanism resulting in trastuzumab resistance (36). Moreover, STAT3 activation also stimulates breast cancer stem-like properties resulting in HER2 overexpression and trastuzumab resistance (193). Hence, targeting JAK/STAT3 pathway or administering IL-6 receptor-targeted antibody should overcome the trastuzumab resistance by reducing the bCSC burden. Additionally, CD47 blockade with trastuzumab also eliminates HER2+ BC cells, overcoming trastuzumab tolerance (194).

#### Lapatinib Resistance

Lapatinib is a reversible and selective receptor tyrosine kinase inhibitor, targeting both epidermal growth factor receptor (EGFR) and HER2 (195). In contrast to trastuzumab, lapatinib blocks kinases’ active ATP-binding site, thus interfering with receptor phosphorylation (**Figure 4G**). However, despite the initial response in HER2-overexpressing BC, acquired resistance to lapatinib turns out to be a frequent event in the course of treatment. Liu et al. have isolated and characterized several lapatinib-resistant HER2/ER+ BC clones from lapatinib-sensitive BT474 cells through chronic exposure to lapatinib. This group has identified that activation of AXL is associated with lapatinib resistance in these resistant BT474 clones (196). Evidence indicates a close association of breast CSCs in exerting lapatinib resistance. In this context, a recent study suggests that miR-205-5p is highly expressed in bCSCs. miR-205-5p represses ERBB/HER receptors in bCSCs, leading to resistance to targeted therapy (197). Silencing miR-205-5p in bCSCs, followed by lapatinib treatment, significantly reduces BC proliferation, resensitizing BC cells toward EGFR/anti-HER2 treatments. Furthermore, knockdown of miR-205-5p by locked oligonucleotides significantly reduces EMT and metastatic potential exerted by bCSCs (198). TGF-β-SMAD3 signaling also contributes to trastuzumab and lapatinib resistance, maintaining CSC phenotype in HER2+ settings (199). CD24 supports the expression of HER2 along with activation of PI3K/AKT signaling, resulting in lapatinib resistance (200). Hence, small-molecule inhibitors of SMAD3, or targeting CD24, can attenuate lapatinib resistance and increase the sensitivity of HER2+ BC cells to lapatinib.

## bCSC-Related miRNA Signature Modulating Stemness and Drug Resistance

The regulation of bCSCs by miRNAs (~less than 25 nucleotides) is emerging as an innovative tool to deal with bCSC-driven drug resistance. Tumor suppressor miRNAs and OncomiRs have been implicated to play an essentially important role in the regulation of bCSC self-renewal, differentiation, tumor initiation, EMT, metastasis, and therapeutic resistance (3, 7, 201). In this section, we will briefly discuss these 2 types of bCSC-related microRNA signature, either suppressing or favoring drug resistance, through their regulation of multiple signaling networks.

### Tumor Suppressor miRs in bCSCs

Several miRNAs, miR-30, miR-34, miR-200 family, miR-223, let-7, and miR-600 have been documented for tumor suppressive function (201). miR-223 is downregulated in CD44+CD24−/low bCSC in TNBC compared with non-CSCs (202). Thus, overexpression of miR-223 sensitizes the TNBC cells to tumor necrosis factor-related apoptosis-inducing ligand (TRAIL)-induced apoptosis (202). miRNA expression profiling indicates that miR-200 family (miR-200a, miR-200b, miR-200c, miR-141, miR-429) is significantly downregulated in bCSCs (203). Overexpression of miR-200c inhibits clonogenicity and tumor-initiation potential of bCSCs, mainly through suppressing Notch signaling and its component JAG1 (201, 204). Similarly, miR-205 and miR-200 families are significantly downregulated in post-EMT metastatic BC, and thus overexpression of the miR200 family prevents TGF-β-induced EMT by negatively regulating both ZEB1 and ZEB2 (205). Let-7 miRNA, downregulated in bCSCs, is mainly engaged in restricting cell-cycle progression, self-renewal, and pluripotency of bCSCs by regulating factors like H-RAS, E2F2, and HMGA2 (206). Let-7 miRNA can block self-renewal of bCSCs in ER+ BC background by targeting the Wnt/β-catenin pathway (207). Similarly, miR-30 negatively regulates the stemness of bCSCs and is significantly downregulated in bCSCs. Hence, overexpression of miR-30 diminishes anoikis resistance and self-renewal potential of bCSCs by directly targeting integrin β3 and ubiquitin-conjugating enzyme 9 (208). Overexpression of miR-600 inhibits bCSC self-renewal and decreases *in vivo* tumorigenicity by inhibiting the Wnt/β-catenin pathway, as it targets the enzyme, SCD1, essential for producing active WNT proteins (209). Therefore, in absence of miR-600, the activated Wnt signaling promotes self-renewal, whereas overexpression of miR-600 induces bCSC differentiation into BC cells. Likewise, miR-34a restricts bCSC stemness and chemoresistance to doxorubicin *via* directly inhibiting the Notch signaling pathway. Notably, miR-34a is downregulated in bCSCs, and hence, overexpression of miR-34a inhibits the Notch signaling pathway, sensitizes bCSCs to paclitaxel, and inhibits BC proliferation, migration, and invasion (210). Similarly, miR-34c has reduced expression in breast CSCs, and overexpressing it significantly interferes with EMT, migration, and self-renewal properties through targeting Notch4 (211).

### Oncogenic miRNAs (OncomiR) in bCSCs

Unlike tumor suppressor miRs, oncomiRs such as miR-21, miR-22, miR-155, miR-181, miR-9, and miR-221/222 cluster, show aberrant expression, and stimulate breast tumor growth, by suppressing apoptotic pathways, allowing proliferation, migration, invasion, and cell-cycle progression (3). Hence, strategies that target oncomiR can effectively block bCSC survival and function. miR-155 stimulates bCSC chemoresistance to doxorubicin by targeting CD44, CD90, and ABCG2, and inhibiting miR-155 resensitizes MDA-MB-231 BC cells to doxorubicin (212). Similarly, miR-181 also offers to be a promising therapeutic target to restrict bCSC function as it stimulates bCSC self-renewal potential and colony-formation properties (213). The miR-181/BRCA1 axis has been suggested to promote bCSC phenotypes in primary BC settings. Interestingly, a positive correlation is found between TGF-β expression level and miR-181/BRCA1 pathway activation in primary breast tumor samples (214). TGF-β pathway promotes bCSC population by inducing miR-181 at the posttranscriptional level and downregulating ATM kinase (215). An upregulated expression of miR-21 is positively correlated with poor prognosis, metastasis, and advanced stages of BC (216). miR-21 stimulates proliferation of BC cells and inhibition of apoptosis *via* suppressing tumor suppressors like PTEN, tropomyosin α1 (TPM1), and programmed cell death protein 4 (PDCD4) (7, 217, 218). Importantly, in BC cells, miR-21 regulates EMT through inhibition of PTEN function *via* p-AKT and p-ERK pathways, and re-expression of miR-21 leads to the acquisition of EMT phenotype in bCSCs with the activation of mesenchymal markers (vimentin, N-cadherin, α-SMA) (219, 220). Another important piece of evidence recognizes miR-22 as a crucial epigenetic modifier, regulating stemness, EMT, and metastasis in BC by silencing TET family-dependent chromatin remodeling (221). Importantly, two other oncomiRs, miR-9 and miR-221, are associated with poor clinical outcomes in BC patients. An enhanced expression of both miR-9 and miR-221 leads to an increase in the SP colonies with CSC-like features, and radically increasing bCSC stemness, migration, and invasion *via* upregulating Oct-4, Nanog, and CD133. However, knockdown of both miR-9 and miR-221 reduced the number of SP colonies and accordingly reduced bCSC self-renewal potency, migration, and invasion (222). Therefore, drugs targeting this class of drug-resistant oncomiRs can resensitize the BC cells to chemotherapies. Recently, MSC-released exosomes, containing specific miRNA sequences, are being utilized for the targeted killing of chemoresistant bCSCs (201, 223).

## Mechanisms and Approaches to Overcome Multimodal Drug Resistance

In this section, we review the recent development of bCSC-targeting therapeutic platforms, based on small-molecule inhibitors, nanotherapeutics, molecules affecting different BC signaling networks, and bCSC-specific immunotherapy for targeting breast cancer-associated multidrug resistance.

### ALDH1 Inhibitors, HIF1α Inhibitors, and EGFR/HER2 Inhibitors

The previously established bCSC marker, ALDH, is a prerequisite for the maintenance of the drug-tolerant breast cancer stem-like population as it protects them from ROS-associated toxic effects (224). Since the ALDHhighCD44+ subpopulation reflects higher metastatic ability both *in vitro* and *in vivo* relative to ALDHlowCD44− and shows resistance to standard cancer therapies, inhibition of ALDH activity through all-trans retinoic acid (ATRA) or diethylaminobenzyldehyde (DEAB) sensitizes this population to treatment (51). ATRA reduces the activity of both ALDH1A1 and ALDH3A1 and stimulates CSC differentiation. Hence, combination therapy of ATRA with a standard chemotherapy regimen could fetch promising results for eliminating bCSCs. The HIF family members, HIF1α and HIF2α are crucial regulators of cancer stemness (135). Mechanistically, HIF1α activates the survival genes in hypoxic conditions, whereas HIF2α interacts with the promoter of Oct4 and Nanog. Hence, HIFs are critical for the chemoresistance exerted by bCSCs (135). This study proposes that the treatment of human BC cells with chemotherapeutic agents such as paclitaxel and gemcitabine leads to survival and enrichment of bCSCs, which in turn depends on the HIFs. Studies involving mice breast tumor models further elaborated that chemotherapy along with HIF inhibitors, such as digoxin (interferes with HIF1α translation) or acriflavine (inhibits dimerization of HIF1α or HIF2α with HIF1β), might improve the survival of BC patients (225–227). Several HIF1 inhibitors including 2-methoxyestradiol, BAY 87-2243, and PX-478 2HCI are, therefore, undergoing clinical trials (228). Moreover, inhibition of the EGFR/HER2 signaling axis by lapatinib blocked the expression of ABC transporter proteins, ABCB1 and ABCG2, which sensitizes MCF-7 tumor spheres to doxorubicin (229).

### Targeting Signaling Pathways in bCSCs

There is an intricate relationship between bCSC maintenance and Notch, PI3K/AKT/mTOR, Wnt/β-catenin, and Hedgehog signaling pathways. The interplay between these signaling pathways also influences the disease outcomes in BC progression. Therefore, targeting these pathways serve as an essential strategy to restrict bCSC expansion and overcome drug resistance phenomena.

#### Notch Signaling

Deregulated Notch signaling in bCSCs represents poor clinical outcomes in drug-resistant BC. Evolutionarily conserved Notch signaling is linked with cell differentiation and cell fate decisions. Notch signaling pathways include 4 receptors (Notch1–4) and 5 ligands such as delta-like ligand (DLL)1, DLL3, DLL4, JAG1, and JAG2 (230, 231). Interaction with the Notch ligand leads to the release of its intracellular domain (NICD), which then translocates to the nucleus and impacts gene expression in association with different transcription factors. Studies have established links between bCSCs, aberrant Notch signaling, and radio-/endocrine-/chemoresistance. A significantly higher expression of activated Notch1 is noted in the culture media of bCSCs, postradiation (66). A substantial induction of the JAG1 ligand is also evident on the surface of nonadherent bCSCs after fractionated radiation (232). Both JAG1 and Notch pathway contributes to chemoresistance in BC metastasizing to bone (233). Moreover, suppression of Notch1 signaling enhances antitumor efficacy of chemotherapy agents *via* reduction of bCSCs in TNBC (234). Notably, Notch ligand DLL1+ quiescent bCSCs drive chemoresistance *via* NFĸB pathway in BC (235), and disease progression in ER+ BC is dependent on DLL1-mediated Notch1 signaling in bCSCs (236). Notch1 ligands, JAG1, and JAG2 are also overexpressed in endocrine-resistant luminal BC, resulting in an increased bCSC activity (237). Therefore, the blockade of Notch signaling is of clinical importance to eradicate resistant bCSCs and offer long-term disease-free survival.

#### PI3K/AKT/mTOR Signaling

PI3K is a family of lipid kinases that phosphorylate phosphatidylinositol (PI) at the intracellular membrane and plasma membranes. An increased PI3K/AKT/mTOR signaling in bCSCs has been documented over the years, contributing to survival, proliferation, metastasis, and drug resistance in BC cells (238, 239). Mutations, specifically in its catalytic domain, p110α, are the most frequent genetic events, affecting around one-third of BC patients. Alterations in the PI3K/AKT/mTOR pathway in bCSCs result in the TAM resistance in ER+ BC (240, 241). The interaction between PI3K and Wnt/β-catenin pathway is responsible for stemness and self-renewal abilities of bCSCs (242). Therefore, small-molecule inhibitors targeting the key players, PI3K, AKT, and mTOR can reverse the drug resistance and self-renewal abilities of breast cancer stem-like cells. Pan-PI3K inhibitors, such as buparlisib and pictilisib (inhibiting p110α/β/γ/δ); PI3K isoform-specific inhibitors such as alpelisib and taselisib (inhibiting p110α and p110α/γ/δ, respectively); AKT inhibitors such as ipatasertib, capivasertib (AZD5363), and vevorisertib (MK-2206); PI3K/AKT dual inhibitor gedatolisib (PF-05212384); and mTOR inhibitors such as everolimus, vistusertib, and sapanisertib are currently available for the treatment of BC (243). B591, a novel PI3K inhibitor, has shown promising results in targeting breast CSCs in the mouse xenograft model, affecting both its self-renewal potential and EMT (244). However, despite substantial preclinical evidence, the innate and acquired resistance has limited the application of this group of inhibitors in BC.

#### Wnt/β-Catenin Signaling

Wnt/β-catenin signaling contributes to self-renewal, migration, and invasion of bCSCs, leading to systematic dissemination in BC. A significantly higher level of Wnt/β-catenin signaling is noted in bCSCs compared with the bulk of the tumor (245). Hence, Wnt/β-catenin signaling serves as a novel target for restricting BC progression. A highly potent small-molecule inhibitor CWP232228 can preferentially inhibit bCSC proliferation *via* antagonizing the binding of β-catenin to T-cell factor (TCF) in the nucleus (246). Another natural product, gomisin M2, downregulates Wnt/β-catenin signaling and inhibits bCSC proliferation, mammosphere formation, and self-renewal (247). Studies indicate multiple interaction points or crosstalk between Notch and Wnt/β-catenin signaling pathways, and thus it is essential to focus on Notch-Wnt synergies in BC progression (248). In a normal mammary setting, in response to Notch ligand DLL1, macrophages express Wnt ligands (Wnt3, Wnt10A, and Wnt16), important for mammary stem cell numbers and activity (249). Thus, the proteins exerting regulatory effects on both these pathways should serve as a novel therapeutic target and targeted in BC. GSK3β is one such protein that regulates β-catenin stability as well as phosphorylates Notch ICD (250).

#### Hedgehog Signaling

The Hh signaling is another novel target in BC since it is frequently upregulated in bCSCs and contributes to CSC self-renewal and stemness maintenance. The cancer-associated fibroblasts (CAFs) within TME support the maintenance of CSC function in breast tumors *via* their regulation of both Wnt/β-catenin and Hh signaling. Briefly, CAFs promote BC progression through proliferation, invasion, matrix remodeling (*via* matrix production and crosslinking, matrix stiffness, force-mediated matrix remodeling), macrophage, and endothelial cell crosstalk (*via* secretion of VEGF, exosomes, HGF production), chemoresistance, and immunosuppression (251–253). Notably, BC shows divergent CAF phenotypes, including FAP-positive (fibroblast-activating protein α1) CAFs driving immunosuppression and resistance to PD-L1 therapy (254). According to Friedman et al., two distinct subpopulations of CAFs (S100A4+ and PDPN+) exist in human breast tumors, where their ratio decides the clinical outcomes across subtypes and is highly correlated with BRCA mutations in TNBC (255). The interaction between the breast cancer cell and fibroblasts also induces the CAF phenotype through activation of Notch signaling (256). Hence, understanding the full repertoire of CAFs and the dynamic changes as breast tumors evolve can improve the precision of treatment and reverse drug resistance. BCSCs secrete the Hedgehog ligand, SHH, which controls CAFs through activation of Hh signaling (257). The CAFs, in turn, secrete some factors that result in the expansion and self-renewal of bCSCs. Therapeutic targeting of CAFs using the inhibitor molecule of Smoothened, the main effector molecule of the Hh pathway, sensitizes TNBC xenograft models to docetaxel (258). Tetraspanin 8 (TSPAN8), a membrane glycoprotein, enhances BC stemness by activating SHH signaling (259). Activation of Hh signaling results in salinomycin resistance in tumor spheres, generated from the MCF-7 cell line (260). However, the inhibition of the Hh pathway by cyclopamine can sensitize the MCF-7 cells to paclitaxel. Therefore, exploring the detailed mechanisms of Hh-driven bCSC signaling can help in the designing of novel drug candidates to reverse BC drug resistance.

### Targeting bCSC Metabolism

Maintenance of a reduced level of ROS through metabolic reprogramming is one of the strategies adopted by bCSCs to avoid oxidative stress, which is attributed to the higher expression of ROS scavengers including glutathione peroxidase, superoxide dismutase, and catalase. There is a close association between ROS levels and bCSC-driven radioresistance. Pharmacological inhibition of ROS scavengers in bCSCs distinctly reduces their clonogenicity potential, resulting in radiosensitization (261). Moreover, ROS generating drugs can target drug-resistant bCSCs through induction of premature senescence (262). To mitigate a higher energy demand of fast-growing tumor cells, bCSCs further reshape their metabolic machinery. BCSCs are metabolically plastic, which allows them to dynamically switch their metabolic state to favor glycolysis or oxidative phosphorylation (OXPHOS). Unlike the non-CSCs that majorly depend on glycolysis, bCSCs can favor either glycolysis or OXPHOS, depending on the niche. The glycolytic switch in CSCs, in general, contributes to stemness. Metabolic switching from OXPHOS to glycolytic phenotype, known as the Warburg effect, is another survival adaptation exhibited by bCSCs, to sustain growth in nutrient-deprived or hypoxic environments (263–265). Since BCL-2 protein is a crucial regulator of mitochondrial respiration, inhibition of BCL-2 prevents OXPHOS (266). This, in turn, reduces the bCSC burden that depends on OXPHOS. Several OXPHOS-targeting compounds, such as atovaquone, arsenic trioxide, and phenformin are undergoing clinical trials for different solid tumors (267, 268). Interestingly, CSCs with metastatic potential follows a distinct metabolic signature. According to Luo et al., metabolic or oxidative stress plays a crucial role concerning bCSCs’ plasticity between quiescent mesenchymal-like (M) state and proliferative epithelial-like (E) state. Oxidative stress produced due to H_2_O_2_, 2DG, and hypoxia regulates the transition from ROS^low^ M-bCSCs into ROS^High^ E-bCSCs (269). Importantly, hexokinase 2, which catalyzes the initial step of glucose metabolism, is a major target of metformin for altering bCSC metabolism. Therefore, exploiting the metabolic switching of bCSCs could essentially provide a novel platform targeting the multidrug-resistant bCSC population.

### Nanotherapeutics Against bCSCs

**Figure 5:**
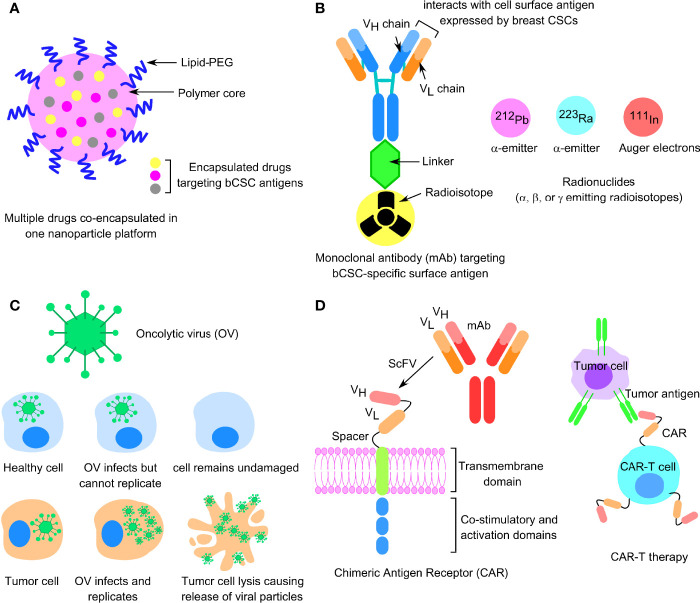
Novel upcoming strategies to reverse bCSC drug resistance. **(A)** Cartoon structure of a nanoparticle-based drug carrier encapsulated with multiple chemotherapeutic drugs targeting bCSC antigens. **(B)** α- or β-emitting radionuclide conjugated with monoclonal antibody targeting breast CSC-specific antigens. **(C)** Design of oncolytic viral particles targeting tumor cells. **(D)** Cartoon representation of chimeric antigen receptor (CAR-T therapy) against CSC surface antigens.

Nanoparticle-based drug carriers (nanocarriers) are often used to specifically deliver chemotherapeutic drugs, siRNAs, miRNAs, and antibodies, designed based on identifying antibodies/aptamers against bCSC-specific markers (**Figure 5A**) (270, 271). Due to the site-specific delivery and improved stability and bioavailability, nanocarriers are appearing as novel platforms for targeting (1) bCSC-specific antigens such as CD44 and ALDH1, (2) drug-efflux ABC transporters (ABCB1 and ABCG2), (3) self-renewing signaling pathways, (4) autophagy process, (5) metabolism, and (6) TME. Sahli et al. developed a triple-drug delivery platform, composed of paclitaxel, verteporfin, and combretastatin (CA4) inside polymer-lipid hybrid nanoparticles to target bCSCs and associated tumor vasculature (272). Gao et al. have further improvised these smart platforms to simultaneously target bCSCs and bulk breast tumor cells by encapsulating the combination of bCSC-specific inhibitor with a chemotherapeutic agent, along with a phytochemical agent or RNA-based therapy (273). HA-modified mesoporous silica nanoparticles loaded with 8 hydroxyquinoline consisting of docetaxel have been designed to eliminate bCSCs (274). This HA modification enables an enhanced uptake of nanoparticles by bCSCs. Another novel chitosan-decorated doxorubicin-encapsulated nanocarrier has been developed to target CD44 surface receptors of bCSCs (275). Recently, a nanocarrier system using PEG-PLA copolymers has been designed for the delivery of autophagy inhibitor molecule, chloroquine, in complex with doxorubicin and docetaxel to eliminate both bCSCs and non-bCSCs (276). Since bCSCs require a specialized niche to survive, nanoparticle-based platforms targeting ECM modifying enzyme lysyl oxidase result in TME disruption (277). A novel HA-based platform encapsulating CD44-targeted docetaxel conjugate is another example of nanocarrier, killing both bCSCs and non-bCSCs (278).

### bCSC-Targeting Strategy Focusing on Immunotherapy

Since CSCs exhibit distinct immune characteristics and express specific immune markers, targeting those molecules as a part of immunotherapy is employed to target CSCs. Different strategies like DC vaccine, adaptive T-cell transfer, oncolytic virus, ICIs, and combination therapies are recent approaches to target bCSCs.

#### DC-Based Vaccine

DCs loaded with CSC lysate or mRNA, administered as vaccines, are capable of eliciting cancer-specific immune responses (279). Notably, DCs are the professional antigen-presenting cells (APCs) that process the antigenic material and present peptide antigens to T cells and activate them. Interestingly, DCs in BC patients exhibit decreased antigen uptake, reduced antigen processing, reduced expression of costimulators, weak migration profile, along decreased IL-12 production (280, 281). Patients with advanced breast and ovarian cancers can be successfully vaccinated by DC loaded with HER2/Neu- or MUC1-derived antigenic peptides (282). Phase-I clinical trial with metastatic BC showed that fusion of breast tumor cells with DCs resulted in immunological and clinical antitumor responses (283). DC pulsed with breast tumor lysate has proved to be a standard method for a source of BC antigen, capable of eliciting anticancer immune responses (284). BCSC-RNA-pulsed DC vaccine also effectively kills breast tumor cells through activation of CD4+ T_h_ lymphocytes and CD8+ cytotoxic T cells. This study highlights the efficacy of bCSC-RNA for priming DC cells in evoking immune response against drug-resistant CSC populations. However, DC-based vaccines present a few drawbacks; they are both cost-effective and time-consuming for patient-specific treatment. Factors like antigenic peptide or CSC-RNA loading on DC, route of administration, and doses are yet to be standardized to resolve these technical limitations.

#### Adoptive T-Cell Therapy

Adoptive T-cell therapy is a personalized mode of immunotherapy to target CSCs. Here, tumor immune lymphocytes (TILs) with intrinsic antitumor activity are isolated from cancer-bearing patients. Following isolation, TILs are cultured in the presence of IL-2 so that they can recognize tumor-associated antigens on cancer cells, and eventually release cytotoxic cytokines, perforin, and granzymes (285). The recent approach focuses on designing CAR-T cells against the CSC surface antigens in different cancer models to achieve complete regression of tumor (286). CARs generally constitute the extracellular binding domain, a single-chain variable fragment (scFv) specific for a tumor antigen, an extracellular spacer domain, a transmembrane domain, followed by an intracellular signaling domain (**Figure 5D**). EGFR-specific CAR-T cells have shown promising results in high EGFR-expressing TNBC cell lines and patient-derived xenograft mouse models (287). Another recent report highlights promising results for HER2-specific second-generation CAR-T therapy for the treatment of breast-to-brain metastasis (288). Other bCSC markers targeted by CAR-T therapy include c-Met, CD133, CD166, CD47, EpCAM, and LGR5 (55, 289, 290). Despite the remarkable clinical success of CAR-T therapy in hematologic cancers, its application is limited in solid tumors. Due to the lack of chemokine expression required for the infiltration of CAR-T cells into the tumor tissues and dense fibrotic matrix in solid tumors, the ability of CAR to get recruited at the tumor site and infiltrate is considerably affected (291). Frequently, CAR-T cells fail to penetrate the tumor tissues through the vascular endothelium (292). Therefore, instead of systemic administration, regional administration of CAR-T cells in solid cancers will be more effective. Altogether, the major limitation of this approach includes burdensome and expensive preparation to isolate the patient-derived T cells and the major side effects resulting from cytokine release syndrome.

#### Oncolytic Viral Therapy

Oncolytic viruses (OVs), a novel class of DNA/RNA-attenuated viruses that selectively infect, replicate inside the tumor cells, and eventually kill them either through modulating the TME or *via* antitumor response (**Figure 5C**) (293). These naturally occurring or genetically engineered viruses have the potential to convert an “immunologically cold” TME into an “immunologically hot” one by increasing the net influx of TILs, consisting of CD4+ and CD8+ T cells, B cells, and NK cells (293, 294). Activated CD8+ cytotoxic T cells and NK cells are associated with a good prognosis, whereas the presence of Foxp3+ Treg cells within the breast TME is associated with a poor prognosis, due to their role in immunosuppression. Immunologically “cold” tumors exhibit a low mutational burden, poor MHC presentation of tumor antigen, poor migration of TILs, and also have reduced expression of PD-L1 on the surface of tumor cells, thus making the response to ICIs inadequate (295). Interestingly, OVs induce a strong antiviral tumor immune response through the production of cytokines like type-1 interferon that in turn promotes PD-L1 expression on tumor cells and also cytokines, such as CCL3 and CCL4, attracting PD-1+ or CTLA-4+ immune cells within TME (296). Eriksson et al. indicated that OVs, Ad5/3-Delta24 and Ad5.pk7-Delta 24, can selectively kill CD44+ CD24−/low bCSC population. Oncolytic herpes simplex virus, oHSV G47Δ, effectively kills bCSCs both *in vitro* and *in vivo*, derived from SK-BR-3 and primary human BC cells (297). A randomized phase II study by Bernstein et al. reported that the combination of oncolytic reovirus (pelareorep) with paclitaxel significantly increased survival of metastatic BC patients (298). Combining pelareoprep with paclitaxel, along with anti-PD-L1 antibody, avelumab (NCT04215146) is presently undergoing phase II clinical study in BC patients (299).

#### Immune Checkpoint Inhibitors

Immune checkpoint ligands such as PD-L1 and PD-L2 are highly expressed on CSCs. The immune cells, on the other hand, express the receptor for these ligands, PD-1. Now, the interaction between PD-1 and PD-L1/PD-L2 interferes with T-cell proliferation and activity, leading to tumor immune suppression, thus serving as a strategy to immune escaping of CSCs (300). Therefore, immune checkpoint blockade of PD-L1/PD-L2 is emerging as a novel therapeutic approach, whereby these CSC-specific ligands are engaged by ICIs, thus making it possible to target CSCs for programmed cell death. Notably, multiple clinical trials on ICIs, targeting CTLA-4, PD-1, and PD-L1 are in progress that are either administered as a single agent or in combination with trastuzumab or with chemotherapeutic drugs, in HER2-enriched and TNBC settings, respectively. In March 2019, FDA has approved the clinical application of anti-PD-L1 antibody, atezolizumab, in combination with nab-paclitaxel, to be administered as the first-line therapy to metastatic or locally advanced PD-L1+ TNBC patients (301). A recent phase Ib clinical study by Nanda et al. explored the antitumor efficacy and safety profile of PD-1 inhibitor molecule pembrolizumab in advanced TNBC patients (302). Furthermore, certain drugs that stimulate PD-L1 degradation can be administered as a combination therapy with ICIs to significantly enhance the efficacy of cancer immunotherapy (303).

## Conclusion and Perspectives

Despite ongoing efforts using novel chemotherapeutics, ICIs, small-molecule inhibitors, or combinations of these innovative therapeutic platforms, bCSC-driven drug resistance remains a public health concern globally. Exploring better bCSC-targeting substitutes is thus the way forward. Radionuclides conjugated with monoclonal antibodies (mAb), administered in radio-immunotherapy (RIT), involve highly potent α- or β-particles to deliver cytotoxic radiation to cancer cells or TME (**Figure 5B**) (304). ^212^Pb-TCMC-trastuzumab using lead-212 (α-particle emitter), is undergoing phase I clinical trial to study its antitumor effects in HER2+ intraperitoneal cancer patients (305, 306). Another isotope, ^111^In-NLS-trastuzumab, is being administered to kill trastuzumab-resistant BC cell lines *via* the emission of Auger electrons (307). Recently, radionuclide therapy using ^223^Ra (α-particle emitter) has been successful in delaying the growth of DTCs in early-stage BC (308). Radioactive iodine therapy with single-domain antibodies targeting HER2 (^131^I-GMIB-anti-HER2-VHH1) documents the first-in-human study, demonstrating the safety profile and efficacy of radionuclide in the treatment of HER2+ BC (309). Importantly, RIT is advantageous in the management of MRD, residual tumor margins following surgery, and CTCs in hematologic malignancy, compared with external beam radiation therapy. Likewise, nanobiotechnology should be fully explored to precisely target bCSC-specific novel antigens, to eliminate the same. The efficacy of synthetic nanoparticles, such as silver (AgNPs) (310), gold (AuNPs) (311), and selenium (SeNPs) (312), has been studied extensively in different types of solid cancers. Notably, AgNPs (313) and AuNPs (314) both have shown encouraging results in BC, although the potency of the same in target killing of breast CSCs is not known. Hence, the potential of this family of radionuclides and nanoparticles should be considered in the targeted killing of bCSCs. In conclusion, cotargeting of multiple signaling networks contributing to bCSC survival and proliferation, by virtue of multimodal targeted therapeutics, will lay the foundation to overcome BC drug resistance.

## Author Contributions

TS has written the manuscript and prepared the figures and table for the manuscript. KEL has edited the manuscript and provided valuable suggestions and inputs in modifying the manuscript. All authors listed have made a substantial, direct, and intellectual contribution to the work and approved it for publication.

## Conflict of Interest

The authors declare that the research was conducted in the absence of any commercial or financial relationships that could be construed as a potential conflict of interest.

## Publisher’s Note

All claims expressed in this article are solely those of the authors and do not necessarily represent those of their affiliated organizations, or those of the publisher, the editors and the reviewers. Any product that may be evaluated in this article, or claim that may be made by its manufacturer, is not guaranteed or endorsed by the publisher.
